# Advancing Standardization in Fragile X Syndrome Molecular Diagnosis: A Dual-Structured and Scoping Review

**DOI:** 10.3390/children13101338

**Published:** 2026-10-01

**Authors:** Bianca Teeny Sallum, Maria Gabriela Custodio de Figueiredo, Monaliza Ehlke Ozorio Haddad, Willian de Souza Santos, Mara L. Cordeiro

**Affiliations:** 1 Escuela de Doctorado, Universidad de Salamanca, 37008 Salamanca, Spain; idu054400@usal.es; 2 Instituto de Pesquisa Pelé Pequeno Príncipe, Curitiba 80250-060, PR, Brazil; gabi.figueiredo@mail.utoronto.ca (M.G.C.d.F.); willian.santos@aluno.fpp.edu.br (W.d.S.S.); 3 Instituto Aprendizagem e Desenvolvimento (IAD), Araucária 83702-080, PR, Brazil; monalizahaddad@iadpr.com.br; 4 Faculdades Pequeno Príncipe, Curitiba 80230-020, PR, Brazil; 5 Department of Psychiatry and Biobehavioral Sciences, David Geffen School of Medicine, University of California Los Angeles, (UCLA), Los Angeles, CA 90095, USA

**Keywords:** fragile X syndrome, *FMR1*, molecular diagnosis, genetic testing, CGG repeat expansion, genetic counseling, diagnostic pathways, scoping review

## Abstract

**Highlights:**

**What are the main findings?**

- Molecular or genomic testing was universally incorporated into Fragile X syndrome diagnosis, but diagnostic workflows varied substantially in their molecular characterization and use of complementary testing.
- Genetic counseling was widely recognized, but documented clinical implementation of counseling, family evaluation, and multidisciplinary management was considerably less consistent.

**What are the implications of the main findings?**

- Standardization should prioritize validated, indication-appropriate molecular workflows and complementary testing when the initial assay does not provide sufficient diagnostic characterization.
- Greater emphasis is needed on translating molecular diagnosis into genetic counseling, family-centered evaluation, and coordinated multidisciplinary care, with future studies reporting these post-diagnostic pathways more systematically.

**Abstract:**

**Background:** Fragile X syndrome (FXS) requires molecular confirmation, yet diagnostic workflows vary in their capacity to characterize expansions and connect diagnosis with subsequent care. This review evaluated alignment between current recommendations and published diagnostic practice. **Methods:** We combined a structured review of recommendations for *FMR1* molecular diagnosis, complementary testing, genetic counseling, and family testing with a scoping review of peer-reviewed primary studies reporting *FMR1* diagnostic or screening workflows relevant to pediatric FXS. PubMed/MEDLINE, Embase, and Scopus were searched without date restrictions. Thirty-four studies were included, with design-specific risk-of-bias assessment and exploratory exact analyses of factors associated with post-initial *FMR1* testing. **Results:** Molecular or genomic testing was reported in all 34 studies, but workflows ranged from conventional PCR and Southern blot to TP-PCR, methylation-sensitive assays, and sequencing-based approaches. Post-initial *FMR1* testing was reported in 22/34 studies (64.7%), with positive-result follow-up documented in 19/34 (55.9%) and follow-up of inconclusive or initially negative results in 14/34 (41.2%). *FMR1* sequencing was uncommon (3/34, 8.8%). Genetic counseling was addressed in 32/34 studies (94.1%), but documented as delivered in only 9/34 (26.5%); family evaluation and multidisciplinary management were also inconsistently reported. **Conclusions:** Published evidence broadly supports molecular *FMR1* testing but shows variability in molecular characterization and downstream care. Standardization should prioritize analytically appropriate workflows, additional testing when required by the initial result or assay limitations, and stronger integration of molecular diagnosis with genetic counseling, family evaluation, and multidisciplinary management.

## 1. Introduction

Fragile X syndrome (FXS) is a leading inherited cause of intellectual disability and an important single-gene cause of autism spectrum disorder (ASD). It is most commonly caused by expansion of the CGG trinucleotide repeat in the 5′ untranslated region of the *FMR1* gene, with more than 200 repeats classified as a full-mutation allele. Full mutations are typically associated with abnormal *FMR1* methylation and reduced or absent production of fragile X messenger ribonucleoprotein (FMRP), a protein with important roles in neuronal and synaptic function [1]. The molecular presentation is nevertheless heterogeneous: individuals may exhibit mosaicism in CGG-repeat size, methylation status, or both, with potential implications for molecular characterization and phenotype [2,3]. Clinical manifestations are similarly variable and may include developmental and language impairments, intellectual disability, behavioral and psychiatric symptoms, seizures, and characteristic physical features [1]. Females with full-mutation alleles generally show greater phenotypic variability and, on average, less severe cognitive and behavioral involvement than males, which may further complicate clinical recognition [4].

Clinical identification of FXS can be challenging because its physical and neurodevelopmental phenotype is neither uniform nor specific. Characteristic physical features may be subtle in young children and become more apparent with age, limiting their value for early recognition [5]. Developmental and behavioral manifestations also overlap with ASD, global developmental delay (GDD), intellectual disability (ID), and other neurodevelopmental conditions, while the autistic phenotype itself varies substantially among individuals with FXS [6]. Accordingly, the diagnostic yield of *FMR1* testing varies with the population and testing strategy. Contemporary studies of broadly investigated ASD, ID, and GDD populations generally report relatively low yields, while clinically selected populations may show higher detection rates [7,8,9]. Diagnostic delay may nevertheless persist when molecular investigation is not incorporated early in the etiological workup. One recent study reported an average interval of 4.63 years between an initial ASD or ID diagnosis and molecular confirmation of FXS [9]. These challenges reinforce the importance of molecular testing for definitive diagnosis rather than reliance on phenotype alone.

Current laboratory recommendations emphasize that *FMR1* testing should adequately characterize CGG-repeat expansions across clinically relevant allele ranges and address additional molecular features when required for interpretation. The American College of Medical Genetics and Genomics (ACMG) and European Molecular Genetics Quality Network (EMQN) provide technical standards for *FMR1* testing and reporting, including appropriate assessment of expanded alleles, methylation and mosaicism where indicated, complementary analysis when the initial assay does not fully resolve the clinically relevant finding or yields an ambiguous result, and clear reporting of assay limitations [10,11]. Molecular confirmation also has implications beyond diagnosis of the index patient. Identification of an *FMR1* expansion can inform genetic counseling, inheritance and reproductive-risk assessment, and evaluation of biologically at-risk relatives [12,13]. Following diagnosis, care may require developmental, behavioral, neurological, and physical surveillance and coordinated multidisciplinary management across the lifespan [12,14].

Despite established recommendations, the extent to which published FXS studies reflect a complete diagnostic and post-diagnostic pathway remains unclear. This two-part review therefore combines a structured synthesis of current recommendations with a scoping review of original published studies. The structured review establishes the reference framework for molecular diagnosis, including the analytical capabilities required for *FMR1* characterization, indications for complementary testing, genetic counseling and family evaluation, and post-diagnostic management. The scoping review then maps the diagnostic approaches reported in primary studies and examines their alignment with this framework, with particular attention to testing methods, post-initial diagnostic testing, molecular characterization, genetic counseling, family evaluation, and multidisciplinary management. By linking recommended practice with the published evidence, this review aims to identify areas of concordance, variation, and persistent gaps across the FXS diagnostic and care pathway.

For this review, a standardized diagnostic pathway was defined as a validated, indication-appropriate workflow that reliably detects and classifies clinically relevant *FMR1* CGG expansions, addresses methylation and mosaicism when indicated, recognizes assay-specific limitations, uses complementary testing for unresolved findings, pursues appropriate investigation when expansion testing is negative despite persistent clinical suspicion, ensures clear molecular reporting, and incorporates genetic counseling and cascade testing where indicated.

### 1.1. Structured Review

A structured narrative review was conducted to define the reference framework for current *FMR1* molecular diagnosis and post-diagnostic care. Searches were conducted between January and August 2026 in PubMed/MEDLINE, Embase, and official professional-society websites using combinations of terms related to “Fragile X syndrome,” “*FMR1* testing,” “CGG-repeat expansion,” “molecular diagnostic guidelines,” “genetic counseling,” and “clinical management.” Eligible documents included current professional guidelines, technical standards, consensus statements, and practice recommendations addressing *FMR1* testing, reporting, complementary investigation, genetic counseling, family evaluation, or post-diagnostic management. Documents were selected on the basis of the authority of the issuing organization, current applicability, and direct relevance to the predefined domains. ACMG and EMQN technical standards formed the core laboratory framework, supplemented by professional recommendations addressing genetic counseling, family testing, and multidisciplinary care. Recommendations were extracted and organized by domain, and areas of agreement, qualification, or difference among documents were synthesized narratively. Because this component was intended to establish a clinically relevant reference framework rather than estimate intervention effects, no quantitative evidence synthesis was performed.

Current recommendations for FXS diagnosis extend beyond detection of an *FMR1* expansion to its appropriate molecular characterization and interpretation within the clinical and familial context. This structured review summarizes the principal recommendations governing molecular diagnosis, complementary testing, genetic counseling and family evaluation, and post-diagnostic management, providing the reference framework for comparison with the studies included in the subsequent scoping review.

### 1.2. Molecular Diagnostic Workflow

Molecular diagnosis of FXS requires direct assessment of the *FMR1* CGG-repeat expansion, as clinical features alone cannot establish or exclude the diagnosis. Alleles are generally classified according to CGG-repeat length as normal (5–44 repeats), intermediate (45–54 repeats), premutation (55–200 repeats), or full mutation (>200 repeats) [10,11,15]. Accurate classification is clinically relevant not only for diagnosis of FXS but also for interpretation of reproductive and familial risk. Full-mutation alleles are typically associated with abnormal *FMR1* methylation and transcriptional silencing, whereas intermediate and premutation alleles do not usually cause FXS. Premutation alleles nevertheless carry reproductive implications because of their potential for expansion during maternal transmission and are associated with other *FMR1*-related conditions, including fragile X-associated primary ovarian insufficiency (FXPOI) and fragile X-associated tremor/ataxia syndrome (FXTAS) [10,15].

Current recommendations do not prescribe a single assay or combination of assays as universally required for *FMR1* diagnosis. Instead, laboratories should use validated methods capable of answering the relevant diagnostic question and should recognize the analytical scope and limitations of each method [10,16]. Conventional PCR can provide high-resolution repeat sizing within its validated amplification range, while triplet-repeat-primed PCR (TP-PCR) facilitates detection of expanded alleles that may not be completely amplified by conventional PCR. TP-PCR can therefore establish the presence of an expansion across a broad repeat range, although precise sizing of large or heterogeneous expansions may require additional or assay-specific approaches [17,18]. The principal analytical capabilities, clinical roles, and limitations of methods used for *FMR1* testing are summarized in Table 1.

Additional molecular characterization is required when the initial assay does not adequately resolve clinically relevant features. Full mutations may exhibit size mosaicism, methylation mosaicism, or both, and low-level expanded alleles can be difficult to detect when shorter alleles amplify preferentially. Mosaicism may also differ between tissues, meaning that peripheral blood findings do not necessarily capture the full molecular heterogeneity of an individual [18,19]. When methylation status or complex mosaicism requires further characterization, validated methylation-sensitive methods may be incorporated. These include methylation-specific PCR, methylation-sensitive TP-PCR, and Southern blot analysis [10,20]. Southern blot historically formed part of a comprehensive diagnostic strategy alongside PCR because it can characterize large expansions, methylation, and mosaic patterns simultaneously. However, it is no longer required for every diagnostic specimen when validated PCR-based and methylation-sensitive methods provide the necessary information; it remains particularly useful for complex, ambiguous, discordant, or unresolved findings [10,11].

In practical terms, assay selection should therefore be guided by the molecular question to be resolved. Conventional PCR is appropriate when accurate sizing within the validated amplification range is required, whereas TP-PCR is particularly useful for detecting expanded alleles that may escape conventional amplification. When methylation status or complex mosaicism requires characterization, methylation-sensitive approaches or Southern blot may be needed, while ambiguous or discordant findings should prompt complementary testing selected according to the unresolved feature.

Diagnostic challenges arise when the molecular result is incomplete, borderline, or discordant with the clinical presentation. Large CGG expansions may fail to amplify by conventional PCR because of the high GC content and length of the expanded allele, potentially producing an apparently single-allele result in females or a non-informative result in males. Low-level size or methylation mosaicism may also escape detection when the analytical sensitivity of the assay is insufficient, while tissue-specific mosaicism may result in peripheral-blood findings that do not fully reflect the molecular pattern in other tissues. Borderline repeat sizes require careful sizing and interpretation, particularly near allele-category boundaries. In these situations, results should not be interpreted in isolation: repeat testing with an alternative or complementary method, methylation analysis, and, where clinically justified, testing of another tissue or investigation for sequence variants or deletions/duplications should be considered when molecular findings remain discordant with a strong clinical suspicion of FXS.

Accordingly, additional testing is not universally required when a validated assay has adequately resolved the clinically relevant molecular finding [10,16]. A second or complementary *FMR1* test should therefore not be interpreted as a universal requirement following every positive result. Complementary testing is most relevant when the initial method cannot establish all clinically necessary characteristics, when repeat classification is uncertain, or when methylation or mosaicism remains unresolved [10,16]. Particular caution may also be required near clinically relevant repeat boundaries, where measurement uncertainty may affect allele classification, and in females with an apparently single normal allele, where an undetected expanded second allele must be excluded by an appropriately validated expansion-detection method [16]. Importantly, the adequacy of a diagnostic workflow depends on whether it resolves the clinically relevant molecular question, rather than simply on whether a second test is performed. Additional testing may serve different purposes, including verification of an initial result, complementary *FMR1* characterization, resolution of an inconclusive finding, or second-line genetic investigation after a negative result.

A negative CGG-expansion result likewise excludes only the expansion mechanism within the validated limits of the assay and does not exclude all pathogenic alterations involving *FMR1*. Rare sequence variants, intragenic deletions, and larger structural alterations may disrupt *FMR1* function without producing the typical CGG expansion [21,22]. When clinical or familial suspicion remains strong after negative expansion testing, complementary investigation may therefore include *FMR1* sequence analysis and deletion–duplication analysis [22]. More broadly, negative *FMR1* expansion testing may warrant investigation of alternative genetic causes of the neurodevelopmental phenotype. Depending on the clinical presentation and previous investigations, this may include chromosomal microarray, neurodevelopmental gene panels, exome sequencing, or genome sequencing [23]. Long-read sequencing may eventually permit more integrated characterization of repeat length and structure, methylation, and mosaicism, but its clinical role remains dependent on platform-specific validation and laboratory implementation [24].

Finally, molecular interpretation depends on transparent laboratory reporting. Reports should identify the analytical method used, define the allele categories applied, and classify the detected result accordingly. Repeat size may also be reported when it can be measured with adequate analytical precision, with ranges or qualifying terms used where exact sizing is not technically supportable. Methylation status and size or methylation mosaicism should be described when assessed and relevant to interpretation [16]. Reports should also communicate relevant assay limitations, particularly because some PCR-based approaches may fail to detect large expanded alleles or may preferentially amplify smaller alleles in females and mosaic individuals, while rare *FMR1* pathogenic variants outside the CGG-repeat region may not be detected by expansion-focused testing [10,16]. Cytogenetic fragile-site analysis, historically used in FXS investigation, is no longer considered an acceptable diagnostic method because of insufficient analytical and clinical sensitivity and specificity and has been superseded by direct molecular analysis of *FMR1* [16].

Thus, the recommended standard for FXS diagnosis is best understood as a validated, indication-appropriate molecular workflow rather than a single mandatory assay combination. Such a workflow should reliably detect and classify clinically relevant *FMR1* CGG expansions, characterize methylation and mosaicism when necessary for interpretation, resolve ambiguous or discordant findings through complementary testing, and prompt appropriate second-line genetic investigation when expansion testing is negative but diagnostic suspicion persists. This approach reflects the methodological heterogeneity of *FMR1* testing: different assays have distinct analytical strengths and limitations, and no single method necessarily characterizes every relevant feature of an *FMR1* full mutation [10,16].

**Table 1:** Analytical Capabilities and Clinical Roles of Methods Used for FMR1 Testing.

Method	CGG-Repeat Detection/Sizing	Methylation/Mosaicism Assessment	Clinical Role	Main Limitation/Complementary Testing
**Conventional PCR with capillary electrophoresis** [10,16]	Detects and sizes alleles within the validated amplification range, particularly normal, intermediate, and premutation alleles	No methylation assessment; size mosaicism involving poorly amplified large expansions may be missed	First-line CGG-repeat sizing	Large expansions may not amplify or size reliably; additional expansion-detection and/or methylation-sensitive testing may be required
**TP-PCR with capillary electrophoresis** [17,25]	Detects expanded alleles across a broad range; sizing capability and accuracy are assay-dependent, with exact sizing of large expansions generally limited	No methylation assessment; detection of size mosaicism is assay-dependent	First-line or complementary expansion detection, including when an expanded allele may fail conventional PCR	Expansion detection may not establish exact size or methylation; complementary characterization may be required
**Methylation-specific PCR-based assays (e.g., mPCR-CE, MS-QMA)** [26,27]	Repeat classification and sizing capabilities are assay-dependent	Assesses methylation; may detect methylation mosaicism above assay-specific thresholds	Complementary methylation characterization alongside CGG-repeat testing	Does not necessarily provide complete repeat classification; complex or discordant findings may require another method
**Southern blot with methylation-sensitive digestion** [11,28,29]	Detects expanded alleles; provides approximate sizing of large expansions	Assesses methylation and size/methylation mosaicism	Complementary/reference method for large or complex expansions, unresolved methylation or mosaicism, discordant findings, and selected prenatal cases	Labor-intensive; requires substantial high-quality DNA and has lower sizing resolution for small repeat differences
**MS-MLPA** [30,31,32]	Does not directly classify or size CGG repeats	Assesses FMR1 promoter methylation; can detect methylation mosaicism above the assay’s detection threshold	Complementary methylation and copy-number analysis	Requires complementary CGG-expansion testing; cannot detect inversions or translocations within the gene
***FMR1* deletion/duplication analysis** [11,31,32]	Does not generally assess CGG-repeat expansions	Assay-dependent; methylation may be assessed by some methods such as MS-MLPA	Considered after a negative expansion result when clinically indicated	Detection depends on the assay and the type/size of structural variant; complementary testing may be required
***FMR1* sequence analysis** [11,22]	Conventional short-read sequencing does not reliably detect long CGG expansions	Limited	Second-line investigation for rare pathogenic SNVs or small indels when expansion/dosage testing is negative but suspicion persists	Does not replace dedicated CGG-expansion testing
**Long-read sequencing** [24,33]	May detect and characterize large CGG expansions, repeat size and structure	May assess methylation and mosaicism, depending on platform and analytical approach	Emerging comprehensive or problem-solving approach	Clinical validation, coverage, bioinformatics, cost, and standardization remain platform-specific
**Fragile-site cytogenetics** [11,16]	Historical method for detecting fragile-site expression	Does not assess FMR1 methylation or CGG-repeat size/methylation mosaicism	Historical method	Not recommended as the primary diagnostic test for FXS; direct molecular FMR1 testing is preferred
**Clinical assessment** [15,34]	Not applicable	Not applicable	Establishes the indication for molecular testing and provides clinical context for interpretation	Cannot confirm or exclude an *FMR1*-related disorder

**Note:** *Analytical performance, including sensitivity, specificity and thresholds for detection of either form of mosaicism, is assay-, platform-, specimen-, and laboratory-validation-specific and should not be generalized to entire method classes. MS-MLPA, methylation-specific multiplex ligation-dependent probe amplification; PCR, polymerase chain reaction; SNV, single-nucleotide variant; TP-PCR, triplet-repeat-primed polymerase chain reaction. Size mosaicism refers to the presence of FMR1 alleles with different CGG-repeat sizes, whereas methylation mosaicism refers to variation in methylation status among alleles or cells.*

### 1.3. Genetic Counseling and Family Testing

It is important to distinguish diagnosis of FXS in the index patient from identification of the broader spectrum of FMR1-related alleles within the family. In a child with suspected FXS, molecular testing primarily aims to identify and characterize a full mutation or another pathogenic FMR1 alteration that explains the clinical presentation. Identification of premutation alleles, by contrast, may have broader implications for cascade testing, reproductive risk assessment, and premutation-associated conditions in relatives. These components are therefore related parts of the diagnostic pathway but address distinct clinical questions.

Molecular diagnosis of an *FMR1*-related disorder has implications beyond the individual tested and should be accompanied by appropriate genetic counseling [15,16,35]. Counseling should address the meaning of the molecular finding, X-linked inheritance, potential clinical implications of full-mutation and premutation alleles, reproductive transmission risk, and the implications of the result for biologically at-risk relatives [15,35]. The proband’s molecular result should be sufficiently characterized to support these discussions, including the CGG-repeat category and size, methylation and mosaicism when clinically relevant, and the nature of any non-expansion pathogenic variant identified [10,16].

Following identification of an affected individual or carrier, targeted cascade testing should be offered to biologically at-risk relatives according to the pedigree and inheritance pattern [10,15]. In families with CGG-repeat expansions, evaluation commonly extends through the maternal lineage and may identify relatives with full-mutation or premutation alleles who were previously unaware of their genetic status [15]. Identification of a premutation is particularly relevant because it may inform reproductive risk as well as risks associated with *FMR1*-related conditions such as FXPOI and FXTAS [10,15]. For individuals considering pregnancy, counseling should explain that the probability of expansion during maternal transmission is strongly related to CGG-repeat size [10] and is further modified by the number and position of AGG interruptions [36,37]. AGG analysis can therefore refine estimates of transmission instability in selected premutation alleles [36,37] but does not constitute confirmation of premutation status.

Reproductive counseling should also address available options according to the individual’s molecular result, reproductive goals, and clinical context. These may include natural conception with prenatal diagnosis or in vitro fertilization with preimplantation genetic testing for monogenic disorders (PGT-M) [38]. Prenatal diagnosis may be performed using chorionic villus sampling or amniocentesis, with appropriate attention to maternal-cell contamination and assay validation for fetal specimens; interpretation of methylation in chorionic villi requires particular caution because *FMR1* methylation may not yet be fully established [10]. When PGT-M is pursued, testing requires prior characterization of the familial *FMR1* alteration, appropriate assay validation, pretest counseling regarding technical and biological limitations, and consideration of confirmatory prenatal testing following establishment of pregnancy [38,39].

Genetic counseling and family evaluation should therefore be regarded as integral components of the FXS diagnostic pathway rather than as consequences separate from molecular diagnosis. An informative *FMR1* result should support interpretation for the individual tested while also enabling appropriate cascade testing, reproductive counseling, and risk assessment for the wider family [15,16,35].

**Figure 1:**
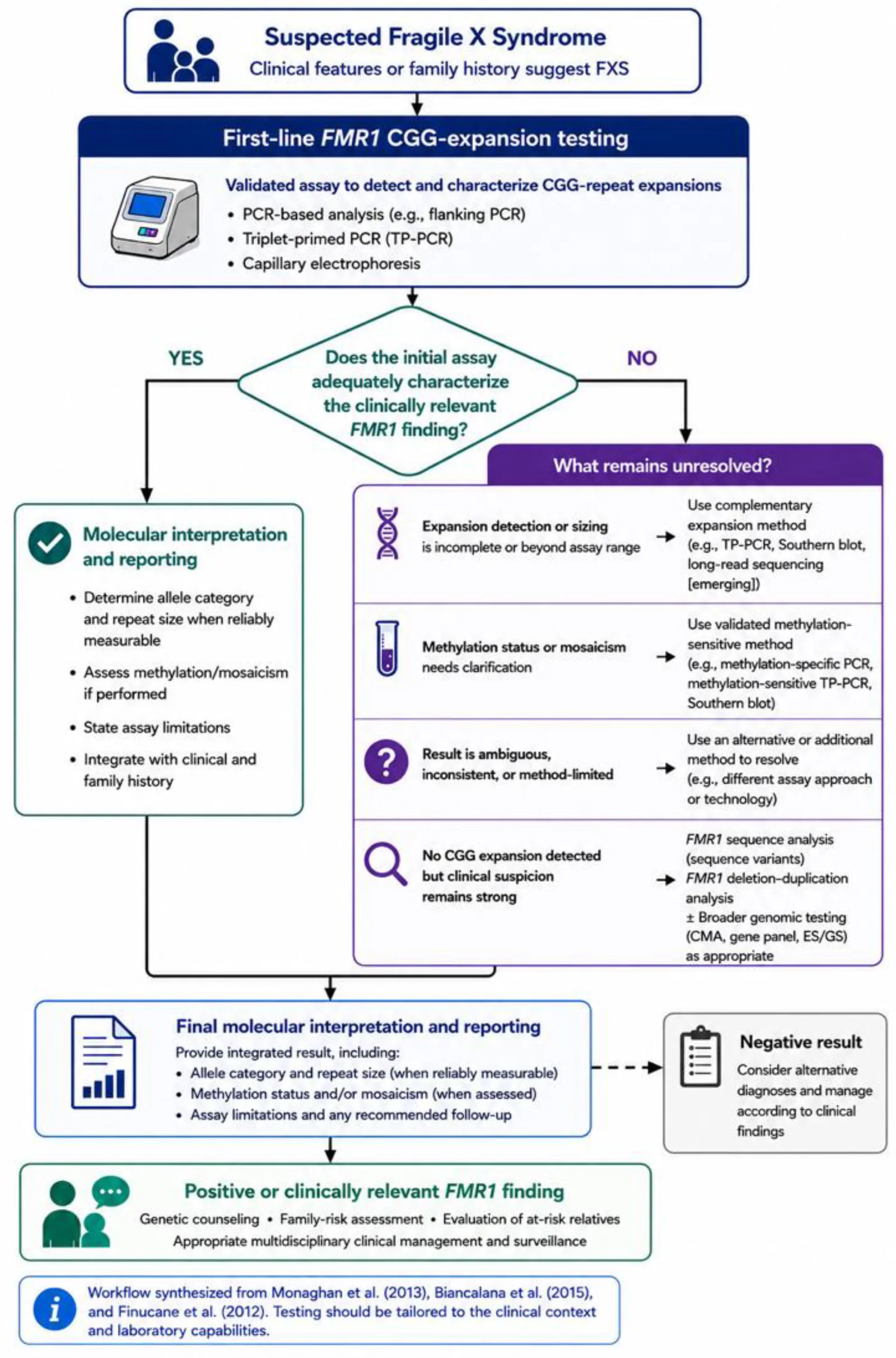
Validated diagnostic workflow for suspected Fragile X syndrome [10,15,16].

Together, these recommendations define a diagnostic and post-diagnostic pathway summarized in Figure 1, which provides the reference framework for evaluating practices identified in the scoping review [10,12,14,15,16,17,22,23,24,35].

## 2. Scoping Review

### 2.1. Materials and Methods

#### 2.1.1. Study Design

This scoping review was conducted according to the methodological framework proposed by Arksey and O’Malley [40] and subsequently refined by Levac et al. [41], with reporting guided by the Preferred Reporting Items for Systematic Reviews and Meta-Analyses extension for Scoping Reviews (PRISMA-ScR) [42]. The review aimed to map the evidence on diagnostic pathways, molecular testing strategies, and management approaches relevant to pediatric Fragile X syndrome (FXS) diagnosis or screening across different healthcare settings and systems. Particular attention was given to FMR1 genetic testing methods, sequencing, post-initial diagnostic testing pathways, testing outcomes, genetic counseling practices, and management recommendations.

A predefined protocol was prospectively registered on the Open Science Framework (OSF; registration: 73txc). Eligibility criteria were structured according to the Population–Concept–Context (PCC) framework. The population of primary interest comprised children and adolescents (0–18 years) with suspected or confirmed FXS and pediatric populations undergoing evaluation for intellectual disability, developmental delay, or autism spectrum disorder. Given the review’s additional focus on FMR1 diagnostic workflows, mixed-age and laboratory-based studies were also eligible when they included an identifiable pediatric component or evaluated molecular testing approaches directly relevant to pediatric FXS diagnosis or screening. Adult family members undergoing cascade testing and prenatal or reproductive samples were retained when they were directly linked to pediatric cases or to the diagnostic workflow under evaluation. Where pediatric and non-pediatric data could not be fully separated, this was documented during data charting and considered in the interpretation of the findings. The concept encompassed FXS diagnostic pathways, FMR1 molecular testing and post-initial diagnostic pathways, genetic counseling, and clinical management. The context included clinical, laboratory, research, public health, educational, and genetic counseling settings across different countries and healthcare systems.

#### 2.1.2. Types of Evidence Sources

Eligible evidence sources comprised primary studies reporting molecular or genetic testing relevant to FXS diagnosis or screening. Study designs included diagnostic and methodological studies involving laboratory method evaluation or validation, cross-sectional studies, cohort studies, retrospective studies, pilot studies, service or health-system studies, case series and case reports, and mixed-method or multi-design studies. Grey literature, including theses and dissertations, was not included.

Studies were excluded if they did not report molecular/genetic testing actually performed in the study population, including publications that only discussed, proposed, or surveyed FXS testing practices. Studies in which FXS classification relied exclusively on a testing approach considered to provide insufficient diagnostic confidence for establishing FMR1 mutation status were also excluded. Review articles were excluded from the evidence synthesis but screened for potentially eligible primary studies and relevant contextual literature. Articles published in English, Portuguese, or Spanish were eligible for inclusion.

#### 2.1.3. Information Sources and Search Strategy

A systematic literature search was conducted in PubMed/MEDLINE, Embase, and Scopus. PubMed and Embase were searched on 6 May 2026, and a supplementary Scopus search was conducted on 19 August 2026 to broaden database coverage. Search strategies combined controlled vocabulary, where applicable, with free-text terms covering five core concepts: FXS/FMR1, diagnosis and screening, genetic testing, management and healthcare access, and pediatric populations. The complete database-specific search strategies are provided in Supplementary Annex SI.

**Figure 2:**
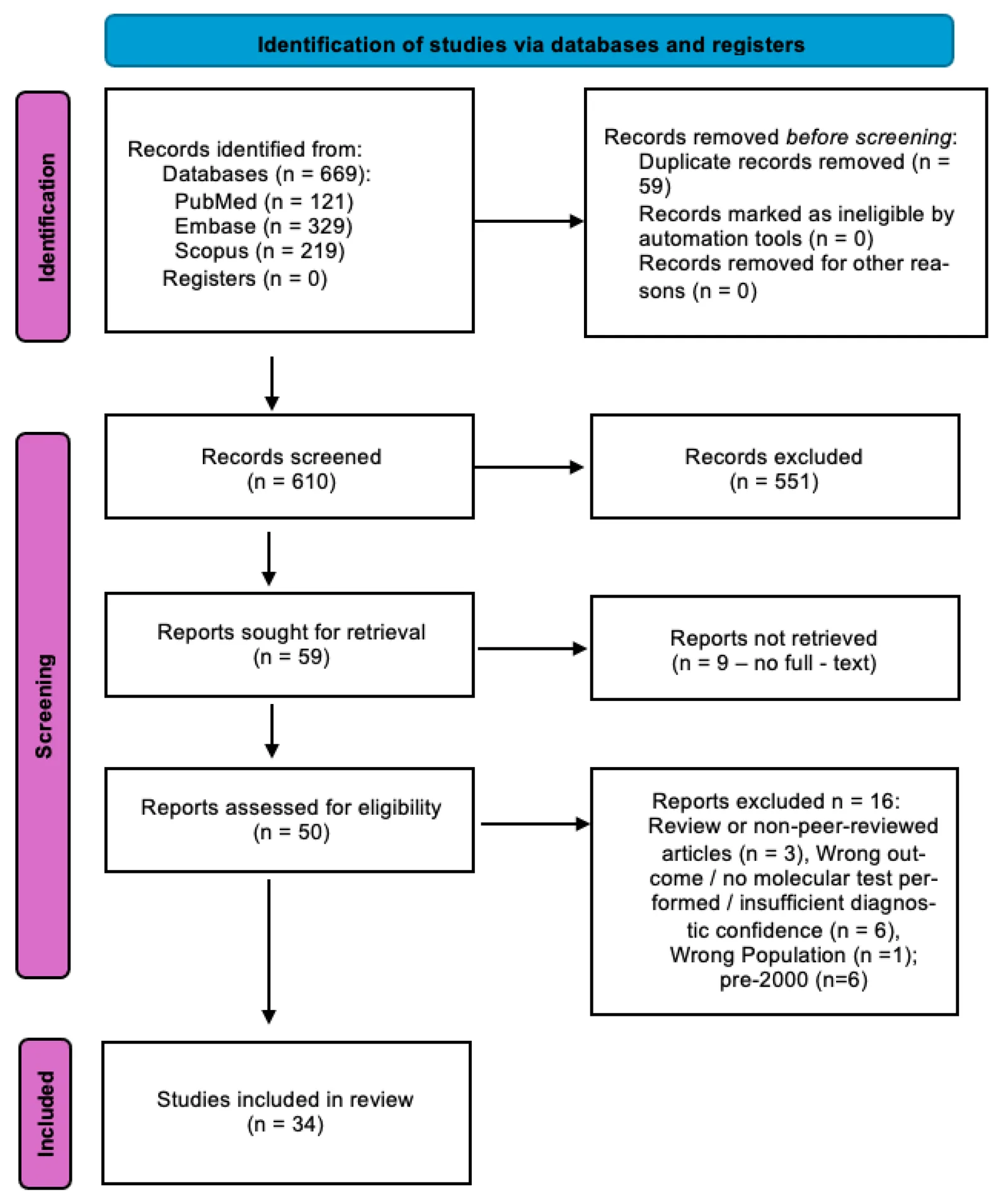
PRISMA-ScR flow diagram of the study selection process.

Records were exported to Zotero (version 90.0.6) and duplicates were removed. Title/abstract screening and subsequent full-text assessment were conducted independently by two reviewers (BTS and MEOH) using Rayyan. Disagreements were resolved by consensus or, when necessary, adjudication by a third reviewer. The study-selection process is presented in the PRISMA-ScR flow diagram (Figure 2).

#### 2.1.4. Data Charting (Data Extraction)

Data were independently extracted by two reviewers using a standardized Microsoft Excel form that was pilot-tested on two studies and refined by consensus. Extracted data covered study design and population characteristics; *FMR1* testing methods and sequencing; post-initial diagnostic testing pathways and molecular outcomes; genetic counseling and management recommendations; and reported study limitations. Genetic counseling was categorized as Clinical Implementation when counseling was explicitly reported as delivered, Recommendation/Benefits when it was recommended or discussed as a benefit without documented delivery, Clinical Data Support when study findings provided counseling-relevant information without reporting implementation, and Not Applicable when genetic counseling was not addressed. Studies in which counseling was both recommended or discussed and supported by counseling-relevant study findings were classified as Recommendation/Clinical Data Support.

Testing data included the initial molecular approach and any subsequent testing, classified according to purpose as verification/confirmation of an initial result, complementary *FMR1* characterization, resolution of an inconclusive or ambiguous result, or second-line genetic investigation after a negative *FMR1* result. Categories were not mutually exclusive, as a single workflow could serve more than one purpose. Follow-up methods, numbers undergoing additional testing, and resulting outcomes were also extracted.

Molecular characterization was mapped through the assays and testing workflows reported by each study; repeat sizing, methylation assessment, and mosaicism assessment were not separately operationalized as binary study-level outcomes. Family evaluation and multidisciplinary management were likewise captured descriptively from reported practices and recommendations rather than as separate implementation outcomes.

Genetic counseling and family evaluation were included because an FXS diagnosis may have implications beyond the index patient, including cascade testing and reproductive risk assessment. These variables were therefore used to characterize their reporting within published diagnostic pathways, rather than to estimate their real-world implementation.

Discrepancies in extraction or classification were resolved through discussion and consensus, with third-reviewer adjudication when required. The final extracted dataset was reviewed for consistency before synthesis.

#### 2.1.5. Risk-of-Bias Assessment

Although risk-of-bias assessment is not mandatory for scoping reviews, it was conducted to support interpretation of the evidence. Assessment was performed independently by two reviewers using design-appropriate tools: JBI checklists for cross-sectional [43], cohort [44], case report [45], and case-series studies [46], and QUADAS-2 [47] for diagnostic accuracy/validation and laboratory/method-comparison studies. For studies with overlapping design features, the tool corresponding to the design most closely aligned with the study’s primary objective and analytical structure was applied. Risk of bias was synthesized at the domain level and reported as low, high, or unclear according to the relevant signaling-question responses; no numerical score or percentage-based overall quality classification was used. Disagreements were resolved by consensus or third-reviewer adjudication. Risk-of-bias findings informed interpretation but were not used for study exclusion. Further details are provided in Supplementary Table S1 and Supplementary Annex SII.

#### 2.1.6. Statistical Analysis

For the exploratory analyses, post-initial FMR1 testing was coded as a binary study-level variable indicating whether any subsequent FMR1-directed testing was reported, irrespective of its purpose; this variable was not interpreted as a measure of diagnostic adequacy or guideline adherence. Examined study-level characteristics included genetic testing method, publication year, journal impact factor, study design, testing setting, management model, diagnostic confidence, healthcare coverage, physician/specialist type, and geographic region.

Exploratory associations between post-initial *FMR1* testing and study-level characteristics were assessed using exact tests in IBM SPSS Statistics, version 32.0.0.0 (134). Fisher’s exact test was used for 2 × 2 contingency tables, and the Fisher–Freeman–Halton exact test for larger contingency tables. All tests were two-sided, with a significance level of *p* < 0.05. Because these analyses were exploratory, no adjustment for multiple comparisons was applied, and findings were interpreted as hypothesis-generating. Analyses were based on available data; journal impact factor was unavailable for three studies.

### 2.2. Results

The search and selection process yielded 34 studies meeting the eligibility criteria for the scoping review. Study and population characteristics are presented in Table 2; genetic and post-initial diagnostic pathways in Table 3; and genetic counseling, management recommendations, and study limitations in Table 4. Risk-of-bias assessment is presented in Supplementary Table S1 and Supplementary Annex SII, while the exploratory appraisal of evidence strength is provided in Supplementary Table S2 and Supplementary Annex SIII. Comparison with previous FXS reviews is presented in Supplementary Table S3.

#### 2.2.1. Study Characteristics

The 34 included studies (Table 2) encompassed diverse methodological approaches, most commonly cross-sectional studies (*n* = 13) and diagnostic or laboratory method-evaluation studies (*n* = 9). The evidence base also included pilot studies, retrospective analyses, service/health-system studies, case series or case reports, one cohort study, and studies combining more than one design or methodological approach.

Studies were conducted across a broad geographical range, including Africa, Asia, Europe, North America, Latin America, and Oceania, with several studies conducted in public healthcare or specialist clinical settings. Study populations ranged from highly selected pediatric cohorts with developmental delay or intellectual disability to newborn-screening populations, family-based cohorts, and large clinical laboratory datasets. Sample sizes consequently varied substantially, from two siblings in a familial case report to more than 100,000 individuals/tests in large-scale screening and laboratory datasets. Accordingly, aggregate study-level frequencies are presented to characterize reporting across the included evidence base and should not be interpreted as estimates of the prevalence of specific diagnostic or post-diagnostic practices across clinical settings.

Most studies included both females and males, although several cohorts were restricted to males, particularly studies evaluating FXS screening in populations with intellectual or developmental disabilities and some newborn-screening programs. Pediatric populations predominated, while some studies additionally included adult relatives, family members, prenatal samples, or broader clinical testing populations when relevant to FXS diagnosis, screening, or cascade testing. Accordingly, findings from the full evidence base are interpreted as informing pediatric FXS diagnostic workflows rather than as estimates derived exclusively from pediatric cohorts.

#### 2.2.2. Risk of Bias of Included Studies

Risk-of-bias concerns varied by study design and were concentrated in specific methodological domains rather than uniformly across studies. Among the 20 studies appraised using the JBI analytical cross-sectional tool, the principal concern was confounding, judged at high risk of bias in 17 of 20 studies, while outcome measurement was at high risk in four and statistical conclusion validity in one. The cohort study showed high risk related to incomplete follow-up. Among case-based studies, concerns primarily involved completeness of inclusion and follow-up. In QUADAS-2 studies, the most frequent concerns involved patient selection and flow and timing, with additional uncertainty regarding reference-standard interpretation in several studies. These findings were used to inform interpretation rather than study eligibility. Detailed domain-level judgments are provided in Supplementary Table S1 and Supplementary Annex SII.

#### 2.2.3. Genetic Testing and Post-Initial Diagnostic Pathways

Considerable heterogeneity was observed in the genetic testing approaches used across the 34 included studies. Testing ranged from conventional PCR or PCR combined with Southern blotting to multi-method workflows incorporating TP-PCR, capillary electrophoresis, methylation-sensitive assays, cytogenetics, and, more recently, sequencing-based approaches. Only 3/34 studies (8.8%) reported FMR1 sequencing, including long-read and whole-genome sequencing approaches. Tested cohorts varied substantially in size, from individual cases and small family-based samples to large-scale laboratory and population-screening cohorts exceeding 100,000 individuals. Accordingly, the proportion of participants identified with premutation or full-mutation alleles varied widely across studies and reflected substantial differences in recruitment populations and study purposes (Table 3).

Post-initial diagnostic testing was heterogeneous in both method and purpose. Rather than treating the performance of a second test as a binary measure of diagnostic adequacy, additional testing was classified according to whether it was used for verification/confirmation of an initial result, complementary FMR1 characterization, resolution of an inconclusive or ambiguous result, or second-line genetic investigation after a negative result. Individual studies could contribute to more than one category. Overall, post-initial FMR1 testing was reported in 22/34 studies (64.7%). Positive-result follow-up was documented in 19/34 (55.9%), while follow-up of inconclusive or initially negative results was reported in 14/34 (41.2%); these categories were not mutually exclusive.

Where the number of participants undergoing additional testing was reported, the extent and purpose of follow-up varied markedly. Some studies retested all initially positive cases, whereas others restricted additional testing to selected positive, ambiguous, or non-amplifying samples. In several studies, post-initial testing produced no additional positive cases, while in three studies (codes 3, 4, and 16) the number of positive results decreased after follow-up assessment. Other studies either did not report the post-initial testing outcome or did not perform a separate post-initial procedure. Detailed testing methods, diagnostic workflows, cohort sizes, PM/FM detection rates, follow-up tests, and post-initial testing outcomes for each study are presented in Table 3.

#### 2.2.4. Genetic Counseling and Management Recommendations

Genetic counseling was addressed in 32 of 34 studies (94.1%), although its role varied across the evidence base. In most studies, counseling was discussed as a recommendation or anticipated benefit of genetic diagnosis, whereas 9 of 34 studies (26.5%) explicitly documented its clinical implementation. Two studies (codes 6 and 20) did not report genetic counseling.

Family evaluation and multidisciplinary management were not separately quantified as implementation outcomes because reporting generally did not distinguish recommendations or referrals from documented delivery of these components.

Management recommendations most frequently emphasized molecular diagnosis and risk stratification, early diagnosis and intervention, and genetic counseling. Other recurrent recommendations included cascade testing or family screening, development or standardization of diagnostic infrastructure, multidisciplinary management, and newborn screening. Several studies combined these approaches, particularly by linking molecular confirmation with genetic counseling and subsequent testing of relatives.

Reported study limitations were dominated by technical or assay-related constraints, followed by limitations in generalizability, missing clinical or family data, small sample sizes, and selection, referral, or participation bias. Some studies reported multiple overlapping limitations, whereas several did not explicitly report study limitations. Study-level genetic counseling status, management recommendations, and reported limitations are summarized in Table 4.

#### 2.2.5. Associations with Post-Initial *FMR1* Testing

Exploratory exact analyses identified an association between post-initial *FMR1* testing and genetic testing method. Post-initial testing differed significantly by genetic testing method (Fisher–Freeman–Halton exact test = 11.274, *p* = 0.005), and was reported in all studies using PCR + Southern blot (7/7, 100%) or conventional PCR only (5/5, 100%), compared with 9/17 (52.9%) using combined workflows (≥2 methods) and 1/5 (20.0%) using other methods. Testing setting showed a borderline nominal association with post-initial testing (Fisher–Freeman–Halton exact test = 7.695, *p* = 0.046), which was reported in all studies conducted in mixed settings (8/8, 100%), compared with 8/13 (61.5%) in clinical settings, 5/9 (55.6%) in research settings, and 1/4 (25.0%) in public/private service settings. No significant associations were observed for year, journal impact factor, study design, management model, diagnostic confidence, healthcare coverage, physician/specialist type, or geographic region (all *p* > 0.05). Given the exploratory nature of these analyses and the absence of adjustment for multiple comparisons, these findings should be interpreted as hypothesis-generating.

#### 2.2.6. Alignment with Guideline Recommendations

Comparison with the recommendations synthesized in the structured review showed broad adoption of molecular genetic testing, but greater variability in the completeness of diagnostic workflows. All included studies used molecular or genomic methods as part of the diagnostic investigation, although the specific approaches ranged from conventional PCR and PCR–Southern blot workflows to TP-PCR, methylation-sensitive assays, chromosomal microarray, and sequencing-based methods. Post-initial testing served different analytical and clinical purposes across the included studies, including verification of an initial result, complementary characterization of *FMR1* repeat size, methylation, or mosaicism, resolution of inconclusive findings, and second-line genetic investigation after a negative result. These functions were therefore considered separately rather than collapsed into a single binary measure based on whether a second test was performed. Importantly, absence of a second test did not necessarily indicate departure from current recommendations, because additional testing is not universally required when the initial validated assay adequately resolves the clinically relevant molecular finding. Reported workflows varied in whether they incorporated methods capable of assessing repeat size, methylation, and mosaicism; these features were not separately quantified as study-level outcomes. FMR1 sequencing or sequencing-based investigation was uncommon (3/34, 8.8%), consistent with its more limited role as a second-line approach for selected cases rather than routine first-line testing.

## 3. Discussion

This review identified a consistent recognition of the central role of molecular testing in Fragile X syndrome (FXS), but also substantial variation in how molecular findings were characterized and translated into subsequent care. Across the 34 included studies, molecular or genomic methods were universally incorporated into diagnostic investigation, although testing strategies ranged from conventional PCR and Southern blot to TP-PCR, methylation-sensitive assays, and sequencing-based approaches. Post-initial *FMR1* testing was reported in 22 studies (64.7%) (Table 3), reflecting substantial heterogeneity in diagnostic workflows. Genetic counseling was also widely recognized, being addressed in 32 of 34 studies (94.1%), whereas evidence of consistent implementation of family evaluation and multidisciplinary management was more variable. Taken together, these findings suggest that the principal gap identified across the published evidence lies not in recognition of the need for molecular diagnosis, but in the consistent documentation and evaluation of how molecular findings are translated into comprehensive diagnostic characterization, genetic counseling, family-centered evaluation, and coordinated clinical management (Table 4).

### 3.1. Diagnostic Workflow and Alignment with Current Recommendations

The universal use of molecular or genomic investigation across the included studies is broadly consistent with current recommendations that FXS diagnosis should rely on direct assessment of *FMR1* rather than clinical phenotype alone. However, the substantial diversity of testing strategies reflects both the long temporal span covered by the literature and the evolution of *FMR1* diagnostics. Accordingly, older studies were interpreted in the context of the standards available at the time of publication and were not considered non-adherent solely because methods or recommendations introduced later were absent. Historically, conventional flanking PCR combined with Southern blot provided complementary information, with PCR permitting repeat sizing within its amplification range and Southern blot enabling detection and characterization of large expansions, methylation, and mosaicism. More recent workflows increasingly incorporate TP-PCR, capillary electrophoresis, and methylation-sensitive PCR approaches, which can reduce reliance on Southern blot when appropriately validated [10,11,16].

Importantly, the presence or absence of a second test was not interpreted as a simple measure of adherence to current recommendations. Post-initial testing served distinct purposes across the included studies: verification of an initial result, complementary *FMR1* characterization, resolution of an inconclusive or ambiguous finding, and second-line genetic investigation after a negative result. These functions address different analytical and clinical questions and were therefore considered separately. Neither ACMG nor EMQN guidance prescribes routine repetition of every positive result using a second complementary method; rather, the diagnostic workflow should adequately characterize the clinically relevant features of the allele and employ additional testing when the initial assay leaves uncertainty regarding repeat classification, methylation, mosaicism, or an unexpected or discordant result [10,11,16]. The greater concern identified in the scoping review is therefore variable completeness of molecular characterization. Several studies used methods that established the presence of an expansion but did not independently assess methylation or mosaicism, whereas others incorporated Southern blot, methylation-sensitive assays, or multiple complementary methods. This distinction is clinically important because TP-PCR can robustly identify expanded alleles [17] but may not precisely size very large expansions or independently establish methylation status [18], while methylation-specific assays likewise do not necessarily provide complete repeat classification [20].

The infrequent use of *FMR1* sequencing should similarly not be interpreted as a major departure from recommended practice. Sequencing-based *FMR1* investigation was reported in only a small proportion of included studies, but CGG-repeat expansion accounts for the great majority of FXS and conventional sequence analysis is not the primary diagnostic approach for detecting large repeat expansions [10]. Current recommendations instead support sequence and deletion–duplication analysis as second-line investigations when expansion testing is negative but the phenotype or family history continues to suggest an *FMR1*-related disorder, because rare pathogenic sequence variants and structural alterations may otherwise remain undetected [22]. More broadly, exome or genome sequencing may form part of the subsequent diagnostic evaluation of children with unexplained developmental delay or intellectual disability when initial targeted testing is unrevealing [23]. Thus, the principal opportunity for greater standardization lies not in requiring a uniform combination of laboratory techniques, but in ensuring that each diagnostic workflow is validated for its intended purpose, characterizes the clinically relevant molecular features, and triggers complementary testing when its analytical scope is insufficient.

### 3.2. Factors Associated with Post-Initial FMR1 Testing

The exploratory analysis provides some indication that variation in post-initial *FMR1* testing may be related more closely to the technical and organizational context of testing than to broader study characteristics. Genetic testing method was associated with post-initial testing (*p* = 0.005), while testing setting showed a borderline nominal association (*p* = 0.046). Publication year, journal impact factor, study design, management model, diagnostic confidence, healthcare coverage, specialist type, and geographic region were not significantly associated with post-initial testing. The association with testing method is clinically plausible, as *FMR1* assays differ in their capacity to detect and size expanded alleles and to characterize methylation and mosaicism [10,16]. More recent technical guidance emphasizes that no single method necessarily characterizes all aspects of an *FMR1* full mutation and that additional testing may serve different purposes, including result verification, complementary *FMR1* characterization, resolution of inconclusive findings, or alternative genetic investigation after a negative result [11]. The nominal association with testing setting may similarly reflect differences in laboratory infrastructure, referral pathways, or access to complementary assays, although these mechanisms were not directly evaluated in the included studies. These findings should therefore be considered hypothesis-generating rather than explanatory, particularly because the analyses were conducted at the study level, involved relatively small categorical groups, and were exploratory without adjustment for multiple comparisons.

### 3.3. Genetic Counseling and Multidisciplinary Management

Genetic counseling emerged as one of the most consistently recognized components of post-diagnostic care, being addressed in 32 of 34 studies (94.1%). However, only 9 studies (26.5%) explicitly documented clinical implementation. Because most included studies were not designed to evaluate post-diagnostic care, this finding primarily indicates a reporting and evidence gap rather than a demonstrated absence of counseling in clinical practice (Table 4). This distinction is particularly important in FXS because molecular findings may have implications beyond the affected individual, including inheritance and reproductive risk, premutation-associated conditions, and targeted testing of biologically at-risk relatives [10,15,35]. The comparatively limited documentation of clinical implementation therefore highlights a gap in the published evidence regarding whether and how genetic counseling is integrated into diagnostic pathways.

A similar distinction applies to clinical management. Current guidance conceptualizes molecular diagnosis as the beginning of an ongoing care pathway rather than an endpoint, supporting developmental surveillance and coordinated management of the neurological, behavioral, physical, educational, and psychosocial manifestations of FXS [12,14]. Yet multidisciplinary management was explicitly represented in only a limited subset of the included studies, while recommendations more commonly emphasized early diagnosis/intervention, molecular diagnosis, genetic counseling, family screening, or improved diagnostic infrastructure (Table 4). This pattern likely reflects, at least partly, the diagnostic and laboratory focus of much of the included literature rather than demonstrating absence of multidisciplinary care in practice. Nevertheless, it highlights a broader evidence gap: the literature provides substantially more information on how FXS is detected than on what happens after the molecular result is established.

### 3.4. Comparison with Previous Reviews

The findings of the present review are broadly consistent with previous reviews of FXS screening, diagnosis, and clinical care. Direct molecular assessment of *FMR1* has consistently been emphasized for establishing the diagnosis, although the specific laboratory approaches described have evolved over time [81,82]. Earlier reviews emphasized PCR and Southern blot, whereas more recent literature incorporated expanded-repeat PCR approaches and situated *FMR1* testing within broader genetic evaluation of neurodevelopmental disorders [81,83]. Across the seven previous reviews examined, these molecular testing recommendations were highly concordant with the current structured-review recommendations (Supplementary Table S3). Recommendations regarding complementary testing were less uniform: post-initial testing and reflex molecular characterization following a positive screen were explicitly described in some reviews [84], whereas others discussed available molecular methods without specifying a comparable reflex-testing algorithm [82,85]. This parallels the heterogeneity observed among the 34 primary studies and supports interpreting variation in post-initial testing pathways in relation to the analytical scope of the initial assay rather than as a simple measure of adherence or non-adherence.

Previous reviews also emphasized genetic counseling, family/cascade testing, and multidisciplinary management, including the reproductive and clinical implications of FMR1 variants for extended families and coordinated care across the lifespan [81,85,86,87] (Supplementary Table S3). The present review extends this literature by examining how these principles are represented in primary studies of diagnostic practice: while molecular testing was universal and genetic counseling was widely acknowledged, documentation of counseling, family evaluation, and multidisciplinary management was considerably less consistent. This distinction between what is recommended and what is documented in practice represents an important contribution of the present synthesis.

### 3.5. Strengths and Limitations

This review has several strengths, including the integration of a structured review of current recommendations with a scoping review of 34 primary studies, allowing recommended diagnostic and management pathways to be compared with their representation in the published evidence. The broad geographical and temporal coverage captured the evolution of *FMR1* testing across diverse populations and healthcare settings, while duplicate screening and extraction, design-specific risk-of-bias assessment, and an exploratory evidence-strength assessment strengthened the transparency of the synthesis. Several limitations should nevertheless be considered. The included studies were highly heterogeneous in design, population, setting, and diagnostic purpose, limiting direct comparison across studies, while much of the evidence originated from selected, referral-based, or specialist populations and may not reflect routine clinical practice. Reporting of post-initial diagnostic testing, genetic counseling, family evaluation, and post-diagnostic management was inconsistent; consequently, absence of reporting could not be interpreted as absence of clinical practice. Furthermore, family evaluation and multidisciplinary management were captured at a broad descriptive level rather than as separately operationalized outcomes. The included studies did not consistently distinguish whether genetic counseling was recommended versus actually delivered, whether cascade testing was offered versus performed, or whether multidisciplinary referral resulted in documented longitudinal follow-up. Consequently, these domains could not be quantified with the same level of detail as molecular testing and complementary diagnostic workflows, and findings related to post-diagnostic care should be interpreted primarily as indicators of reporting patterns rather than direct measures of clinical implementation.

The exploratory analyses of factors associated with post-initial FMR1 testing were conducted at the study level, involved small categorical groups, and were not adjusted for multiple comparisons; they should therefore be interpreted as hypothesis-generating. Finally, restriction to peer-reviewed publications in English, Portuguese, and Spanish and exclusion of grey literature may have omitted relevant evidence, particularly from settings where FXS diagnostic practices are less frequently represented in indexed journals.

### 3.6. Implications and Future Directions

The findings have implications for both clinical practice and future research. Standardization of FXS diagnosis should focus less on prescribing a single combination of laboratory methods and more on ensuring that testing workflows adequately characterize clinically relevant *FMR1* findings and incorporate complementary testing when the analytical scope of the initial assay is insufficient. Molecular diagnosis should also be more consistently connected to genetic counseling, evaluation of at-risk relatives, and multidisciplinary follow-up. Future studies should therefore move beyond reporting whether these components are recommended and document their actual implementation within diagnostic and post-diagnostic pathways. Prospective and implementation-focused studies are needed to evaluate how these pathways operate across different healthcare settings, including their feasibility and cost-effectiveness. Continued evaluation of emerging sequencing technologies may also clarify their role in selected unresolved cases and determine how they can complement established repeat-expansion and methylation-based approaches.

## 4. Conclusions

Molecular testing is firmly established as the basis of FXS diagnosis, but considerable heterogeneity remains in the completeness of molecular characterization and in the transition from diagnosis to subsequent care. Current recommendations support flexible, analytically appropriate diagnostic workflows rather than a universal requirement for a particular combination of assays or routine additional testing. The evidence also suggests limited and inconsistent reporting of post-diagnostic care, including genetic counseling, family evaluation, and multidisciplinary management, although the available studies were not designed to quantify implementation of these components. Future efforts should therefore prioritize robust and appropriately characterized molecular diagnosis while improving the explicit documentation and evaluation of post-diagnostic pathways, including genetic counseling, family evaluation, and multidisciplinary care.

## Figures and Tables

**Table 2:** Study and population characteristics.

Code	Study ID	Study Design	Objective(s)	Population	Sample Size (N) + Analytic N	Inclusion Criteria	Sample Characteristics
1	Alliende et al., 2008 [48]	Cross-sectional	Identify genetic and non-genetic causes of ID using a genetic screening protocol.	Students with non-Down syndrome ID in special education schools in Chile.	Initial N = 133; included N = 103; genetic screening N = 92.	Age 5–24 years; non-Down syndrome ID; informed consent.	Public healthcare; mixed/specialist healthcare professionals; females and males.
2	Au et al., 2025 [8]	Retrospective, Cross-sectional	Evaluate FXS diagnostic yield, pathways, and clinical characteristics in Hong Kong.	Chinese individuals undergoing FXS testing for DD, ID, or ASD in Hong Kong.	FXS testing N = 7291; molecularly confirmed FXS N = 103 (61 families); clinical analysis N = 70.	FXS testing for neurodevelopmental disorders; confirmed full mutation for phenotype analysis.	Mixed coverage; mixed healthcare professionals; females and males.
3	Bailey et al., 2017 [49]	Cohort, Pilot Study, Service/Health System Study	Assess feasibility, outcomes, and potential harms of voluntary FXS newborn screening.	Newborns and families participating in voluntary FXS screening in the United States.	N > 28,000 families approached; analytic N > 17,000 newborns screened.	Parental consent for voluntary dried blood spot FXS newborn screening.	Mixed healthcare professionals; diverse population; females and males; income and maternal education recorded.
4	Bhowmik et al., 2009 [50]	Cross-sectional	Determine FXS prevalence among neurobehavioral outpatients using molecular/protein screening.	Indian neurobehavioral outpatients with developmental/neurological disorders.	N = 157; analytic N = 157.	Developmental/neurological disorder diagnosed according to DSM-IV criteria.	Specialist healthcare professionals; females and males.
5	Chow et al., 2003 [51]	Cross-sectional, Pilot Study	Evaluate dried blood spot PCR screening for FXS in newborn Taiwanese boys.	Newborn boys undergoing FXS screening in Taiwan.	N = 4843; analytic N = 4843.	Newborn boys enrolled through participating screening centers.	Specialist healthcare professionals; males only.
6	Christofolini et al., 2007 [52]	Diagnostic Accuracy/Validation	Evaluate FXS clinical checklists against molecular diagnosis in Brazilian males with ID.	Brazilian males with ID attending a tertiary outpatient clinic.	N = 200; analytic N = 200.	Male with ID and normal karyotype.	Specialist healthcare professionals; males only.
7	Cotter et al., 2016 [53]	Retrospective, Cross-sectional, Service/Health System Study	Audit FMR1 testing patterns and post-test referrals in Victoria, Australia.	Individuals undergoing FMR1 testing in Victoria, Australia.	N = 19,861 tests; analytic N = 12,251. 2014 comparison N = 2423.	Diagnostic/carrier FMR1 tests with recorded date of birth.	Mixed coverage; mixed healthcare professionals; females and males.
8	Dave & Shetty, 2010 [54]	Laboratory/Method comparison	Evaluate PCR-based FXS screening for counseling, prenatal diagnosis, and family management.	Indian children with ID/DD and suspected FXS.	Initial screening N = 720; cytogenetic evaluation N = 443; molecular analysis N = 65.	ID/DD with clinical suspicion of FXS or unexplained ID.	Specialist healthcare professionals; females and males.
9	Fatima et al., 2014 [55]	Cross-sectional	Estimate FMR1 full-mutation frequency/CGG distribution and establish an FXS testing protocol.	Pakistani children aged 5–18 years with unexplained ID and community controls.	N = 583; ID N = 333; controls N = 250. Analytic N = 583 for CGG polymorphism; N = 333 for FXS/full-mutation analysis.	Unexplained ID, age 5–18 years; cerebral palsy/Down syndrome excluded; unaffected community controls.	Special-school/community setting; ages 5–18; females and males (ID: 68.7% male; controls: 52.8% male).
10	Garrido-Torres et al., 2024 [56]	Retrospective, Cross-sectional	Evaluate genetic testing utilization and abnormalities among children with ASD.	Children with ASD receiving tertiary mental healthcare in Spain.	N = 440; analytic N = 440.	Confirmed ASD with tertiary mental-health follow-up.	Public healthcare; mixed healthcare professionals; females and males; socioeconomic variability reported.
11	Gigonzac et al., 2016 [57]	Laboratory/Method Comparison	Standardize capillary electrophoresis-based FMR1 analysis for public-sector FXS diagnosis.	Brazilian patients with ID suggestive of FXS.	N = 26; analytic N = 26.	ID with referral for FMR1 investigation.	Public healthcare; females and males.
12	González-del Angel et al., 2000 [58]	Cross-sectional	Assess FMR1/FMR2 expansions in Mexican children with unexplained ID.	Mexican children with unexplained ID referred to a tertiary pediatric genetics service.	N = 62 unrelated index cases; analytic N = 62.	Unexplained ID after clinical, cytogenetic, metabolic, and neuroimaging evaluation.	Tertiary pediatric hospital; ages 13 months–17 years (mean 6.7); 53 males, 9 females.
13	Hnoonual et al., 2025 [59]	Cross-sectional	Characterize 30 years of FXS molecular diagnosis, frequency, and testing workflows in Thailand.	Thai individuals with ID, DD, ASD, or referral for FXS testing.	N = 1480; analytic N = 1480.	ID, DD, ASD, or referral for FXS DNA testing.	Mixed coverage; specialist healthcare professionals; females and males.
14	Hosny Omar et al., 2016 [60]	Cross-sectional	Determine FMR1 mutation frequency and evaluate PCR screening in Egyptian males with ID.	Egyptian males with idiopathic ID.	N = 64; analytic N = 64.	Idiopathic ID without identified syndromic, chromosomal, metabolic, neurological, or acquired cause.	Specialist healthcare professionals; males only.
15	Hou et al., 2023 [61]	Laboratory/Method Comparison	Validate long-read sequencing for comprehensive FMR1 analysis against conventional testing.	Individuals with suspected FXS referred for genetic evaluation in China.	N = 238 subjects from 234 families; analytic N = 238.	Reproductive indication, neurodevelopmental disorder, suggestive features, or FXS family history.	Specialist healthcare professionals; females and males.
16	Ibañez et al., 2022 [62]	Diagnostic Accuracy/Validation	Evaluate WGS for detecting repeat expansions and diagnosing neurological disorders.	Individuals with suspected neurological repeat-expansion disorders in the UK.	Diagnostic cohort N = 404; clinical validation cohort N = 11,631; analytic N = 11,631.	Suspected neurological repeat-expansion disorder after standard NHS genetic testing.	Public healthcare; mixed healthcare professionals; females and males.
17	Jain et al., 2013 [63]	Cross-sectional	Characterize ID etiology in children using a structured diagnostic algorithm.	Children with ID attending a tertiary care center in India.	N = 142 identified; analytic N = 101.	Age 3 months–12 years with ID, DD, autism, or neuro-regression; previously diagnosed cases excluded.	Public healthcare; specialist healthcare professionals; females and males.
18	Kadam et al., 2018 [64]	Case report	Report familial FXS in two siblings without prior family history.	Two Indian siblings with molecularly confirmed FXS.	N = 2 siblings; analytic N = 2.	Clinical suspicion of FXS with molecular confirmation.	Specialist healthcare professionals; females and males.
19	Kamga et al., 2020 [65]	Cross-sectional	Describe cascade counseling and FMR1 testing in an affected Cameroonian family.	Members of an extended Cameroonian family affected by FXS.	N = 46; analytic N = 46.	Consenting relatives of molecularly confirmed FXS probands.	Mixed healthcare professionals; females and males.
20	Kraan et al., 2018 [66]	Cross-sectional	Compare FMR1 premutation/gray-zone frequencies in developmental-delay and population cohorts.	Pediatric DD referrals and newborn/population comparison cohorts.	Total N = 35,322 (DD referrals = 19,076; newborn screening = 1997; carrier screening = 14,249).	Pediatric DD referrals (≤18 years); newborn and carrier-screening comparison cohorts.	Females and males; private fee-for-service carrier-screening cohort.
21	Meguid et al., 2014 [67]	Laboratory/Method Comparison	Evaluate rapid PCR for FMR1 premutation detection and family screening.	Egyptian males with suspected FXS and female first-degree relatives.	Total N = 111; analytic N = 85 suspected/family samples.	Males meeting clinical FXS criteria and first-degree female relatives of positive cases.	Specialist healthcare professionals; females and males.
22	Penteado et al., 2017 [68]	Retrospective, Cross-sectional	Evaluate FMR1 molecular testing in suspected FXS within the Goiás public health system.	Individuals with ID and suspected FXS referred through the Goiás public health system.	N = 31; analytic N = 31.	ID with clinical indication/suspicion of FXS referred for molecular testing.	Public healthcare; ages 4–41 years (mean 13); 64.5% male; 25/31 aged 4–19 years.
23	Pozo-Palacios et al., 2021 [69]	Cross-sectional	Describe FXS molecular screening and screened patients in Ecuador.	Ecuadorian males referred for suspected FXS.	N = 247; analytic N = 247.	Male with ID, ASD, language delay, or suggestive family history.	Public healthcare; males only.
24	Rajan-Babu et al., 2022 [70]	Diagnostic Accuracy/Validation, Laboratory/Method comparison	Develop and validate methylation-specific TP-PCR for FMR1 expansion and methylation detection.	Reference and patient samples spanning FMR1 genotype categories.	N = 131 (20 reference + 111 patient samples); analytic N = 109.	Known FMR1 genotype spanning normal, intermediate, PM, or FM ranges.	Multinational; specialist healthcare professionals; females and males.
25	Rauch et al., 2006 [71]	Laboratory/Method comparison	Compare genetic diagnostic yields in unexplained DD/ID and propose an optimized workflow.	Individuals with unexplained DD/ID referred for genetic evaluation in Germany.	Cytogenetic cohort N = 601; genetics clinic cohort N = 569; combined analytic N = 670.	Unexplained DD/ID referred for genetic evaluation.	Mixed healthcare professionals; females and males.
26	Rifé et al., 2002 [72]	Cross-sectional, Pilot Study	Compare PCR and FMRP immunodetection for newborn FXS screening.	Newborn boys undergoing FXS screening in Spain.	N = 200 newborn males; analytic N = 194 dried blood spots and 200 blood smears.	Newborn boys enrolled in Catalonian screening with parental consent.	Public healthcare; mixed healthcare professionals; males only.
27	Roche-Martínez et al., 2025 [73]	Case-series	Assess updated FMR1 testing for diagnosis, prognosis, counseling, and follow-up of atypical FXS.	Individuals with atypical FXS presentations undergoing molecular reassessment in Spain.	N = 9 family members with molecular data; updated molecular diagnosis N = 7.	Previous FMR1 diagnosis with atypical/mild FXS presentation requiring reassessment.	Mixed coverage; specialist healthcare professionals; females and males; predominantly high SES.
28	Ruangdaraganon et al., 2000 [74]	Cross-sectional	Estimate FXS prevalence and characterize affected Thai children with DD/ID.	Thai children with unexplained DD/ID attending a child development clinic.	Eligible N = 129; enrolled/screened N = 114; analytic N = 114. Additional family testing identified 4 affected relatives.	Global DD (≥2 domains) or ID (IQ < 70), full-term birth, and unknown etiology.	Specialist pediatric clinic; ages <3–14 years; 82.5% male; 90.4% with severe DD/ID.
29	Shou et al., 2025 [75]	Laboratory/Method comparison, Diagnostic Accuracy/Validation	Validate a four-primer PCR/CE assay for FMR1 CGG-repeat detection and sizing.	Individuals referred for FMR1 testing across six Chinese centers.	N = 1717 screened; analytic N = 1690.	Suspected FXS/ID, reproductive indication, family history, or genetic counseling referral.	Mixed coverage; mixed/specialist healthcare professionals; females and males.
30	Sofocleous et al., 2008 [76]	Retrospective, Cross-sectional	Characterize 10 years of FMR1 testing, frequency, referrals, and CGG-repeat distributions.	Individuals referred for FMR1 testing in Greece.	Total N = 3888 (patients = 1755; family members = 1733; controls = 400).	ID of unknown etiology after exclusion of other chromosomal abnormalities.	Specialist healthcare professionals; females and males.
31	Sorensen et al., 2013 [77]	Case-series, Pilot Study	Evaluate feasibility, benefits, and risks of FMR1 newborn and cascade screening.	Newborns undergoing FMR1 screening and relatives undergoing cascade testing.	N = 3042 newborns screened; cascade testing N = 44 relatives.	Parental consent for newborn FMR1 screening; screen-positive cases eligible for confirmatory/cascade testing.	Specialist healthcare professionals; females and males.
32	Stoyanova et al., 2022 [78]	Cross-sectional	Evaluate selective FMR1 screening in high-risk children with unexplained ID/DD/autistic behavior.	Bulgarian children with unexplained ID, DD, and/or behavioral problems.	Primary pediatric cohort N = 52; total genetically tested N = 54 including 2 cascade-tested mothers.	Unknown-cause ID/DD/behavioral problems with DD, ID, or autistic-like behavior; known genetic etiologies excluded.	Hospital/genetic counseling setting; ages 2–15 years (mean 6); 86.5% male; 84.0% recruited from pediatric clinics.
33	Strom et al., 2007 [79]	Retrospective, Cross-sectional, Service/Health System Study	Characterize FMR1 testing patterns, allele distributions, and counseling-relevant findings.	Individuals undergoing postnatal or prenatal FMR1 testing in a U.S. clinical laboratory.	Postnatal N = 119,232; prenatal N = 307.	Consecutive postnatal/prenatal FMR1 tests performed during 1992–2006.	Private healthcare; mixed healthcare professionals; females and males.
34	Tzeng et al., 2001 [80]	Diagnostic Accuracy/Validation	Develop low-cost PCR- and Southern blot-based FXS screening strategies.	Individuals with ID, primarily children/youth, in Taiwan.	N = 471 screened; additional blood spot cohort N = 104.	ID, predominantly age < 12 years.	Mixed coverage; specialist healthcare professionals; females and males.

**Note:** *Pediatric populations were the primary population of interest. Mixed-age, family/cascade-testing, prenatal/reproductive, and broader laboratory cohorts were retained when they included a pediatric component or evaluated FMR1 diagnostic workflows directly relevant to pediatric FXS diagnosis or screening. Population composition and the inclusion of non-pediatric participants or samples are specified in the Population, Inclusion Criteria, and Sample Characteristics columns.*

**Table 3:** Genetic testing and post-initial diagnostic pathways.

Code	Genetic Testing Method	FMR1 Sequencing	Post-Initial Testing Workflow	Inconclusive/Negative-Result Follow-Up	Positive-Result Follow-Up	Tested Cohort (N)	PM/FM Positive, n (%)	PM/FM Negative (%)	Follow-Up Test After Initial Result	Additional/Advanced Testing (n)	Post-Initial Testing Outcome
1	PCR + Southern blot	N	PCR in males → Southern blot for abnormal/inconclusive male results and all females.	Y	N	92	3 (3.3%)	96.7%	Southern blot	Not reported	Not reported
2	Combined workflow (≥2 methods), conventional PCR, TP-PCR, Southern blot, methylation-specific PCR	N	Pre-2014: PCR → Southern blot; from 2014: PCR → TP-PCR ± methylation assay.	Y	Y	7291 + cascade-tested family members.	103 (% not reported)	Not reported	Southern blot, TP-PCR, methylation assay	Not reported	Not reported
3	Conventional PCR	N	Dried blood spot PCR → whole-blood PCR for screen-positive infants.	N	Y	17,000	46 (0.3%)	99.7%	PCR	30	Fewer positives after confirmation
4	Combined workflow (≥2 methods), conventional PCR, methylation-specific PCR, cytogenetics, other	N	FMRP immunocytochemistry + PCR → MS-PCR for low FMRP, PM-range, or PCR-failure results.	Y	Y	157	30 (19.1%)	80.9%	Methylation assay	80	Fewer positives after confirmation
5	Southern blot, conventional PCR, combined workflow (≥2 methods)	N	Blood spot PCR → Southern blot for >54 CGG repeats or repeated PCR failure; confirmation not completed.	N	N	4843	4 (0.1%)	99.9%	None	Not applicable	Not applicable
6	PCR + Southern blot	N	PCR → Southern blot for FMR1-positive results.	N	Y	200	19 (9.5%)	90.5%	Southern blot	19	No additional positives
7	PCR + Southern blot	N	PCR → Southern blot for inconclusive or PM/FM results.	Y	Y	12,251	453 (3.7%)	96.3%	Southern blot	Not reported	Not reported
8	Combined workflow (≥2 methods), Southern blot, conventional PCR, cytogenetics	N	PCR → Southern blot for positive/no-amplification results.	Y	Y	65	12 (18.5%)	81.5%	Southern blot	12	No additional positives
9	Conventional PCR	Y	Conventional PCR → sequencing verification; PCR failure → methylation assay → Southern blot.	Y	Y	333	16 (4.8%)	95.2%	Methylation assay, Southern blot, genetic sequencing—other	16	No additional positives
10	Other (FMR1 CGG-repeat testing; assay not specified)	N	No post-initial FMR1 test reported	N	N	142	4 (2.8%)	97.2%	None	Not applicable	Not applicable
11	TP-PCR + capillary electrophoresis	N	No post-initial FMR1 test reported	N	N	26	3 (11.5%)	88.5%	None	Not applicable	Not applicable
12	Conventional PCR	N	PCR in males → Southern blot for all index cases; females underwent Southern blot.	Y	Y	62	2 (3.2%)	96.8%	Southern blot	62	No additional positives
13	Conventional PCR, combined workflow (≥2 methods), Southern blot, methylation-specific TP-PCR + capillary electrophoresis (msTP-PCR/CE), cytogenetics, methylation-specific PCR, TP-PCR + capillary electrophoresis	N	PCR → MS-PCR/TP-PCR/msTP-PCR for failed/ambiguous results → Southern blot for positive/ambiguous results.	Y	Y	1480	103 (7.0%)	93.0%	Methylation assay, TP-PCR, Southern blot	Not reported	Not reported
14	Combined workflow (≥2 methods), conventional PCR, other	N	No post-initial FMR1 test reported	N	N	64	14 (21.9%)	78.1%	None	Not applicable	Not applicable
15	Combined workflow (≥2 methods), conventional PCR, genetic sequencing: long-read FMR1 sequencing (PacBio SMRT/LRS)	Y	PCR and CAFXS long-read sequencing compared in parallel, not sequentially.	N	N	238	9 (3.8%)	96.2%	None	Not applicable	Not applicable
16	Combined workflow (≥2 methods), conventional PCR, genetic sequencing (whole-genome sequencing, WGS)	Y	WGS → visual inspection of expansion calls → PCR confirmation.	N	Y	6731	124 (1.8%)	98.2%	Visual Inspection, PCR	124	Fewer positives after confirmation
17	Combined workflow (≥2 methods), conventional PCR, cytogenetics, MS-MLPA, other	N	No post-initial FMR1 test reported	N	N	4	1 (25.0%)	75.0%	None	Not applicable	Not applicable
18	Combined workflow (≥2 methods), TP-PCR, conventional PCR	N	No post-initial FMR1 test reported	N	N	2	2 (100.0%)	0.0%	None	Not applicable	Not applicable
19	TP-PCR + capillary electrophoresis	N	No post-initial FMR1 test reported	N	N	46	18 (39.1%)	60.9%	None	Not applicable	Not applicable
20	PCR + Southern blot	N	PCR → Southern blot for PM-range or inconclusive results.	Y	Y	19,076	221 (1.2%)	98.8%	Southern blot	Not reported	Not reported
21	Combined workflow (≥2 methods), conventional PCR, other	N	No post-initial FMR1 test reported	N	N	85	30 (35.3%)	64.7%	None	Not applicable	Not applicable
22	TP-PCR + capillary electrophoresis	N	No post-initial FMR1 test reported	N	N	31	6 (19.4%)	80.6%	None	0	Not applicable
23	Conventional PCR	N	Endpoint PCR → real-time PCR for positive/suspected expansions.	N	Y	247	22 (8.9%)	91.1%	PCR	22	No additional positives
24	TP-PCR, methylation-specific PCR, methylation-specific TP-PCR + capillary electrophoresis (msTP-PCR/CE), combined workflow (≥2 methods)	N	No post-initial FMR1 test reported	N	N	111	55 (49.5%)	50.5%	None	Not applicable	Not applicable
25	Combined workflow (≥2 methods), cytogenetics, conventional PCR, methylation-specific PCR, other	N	No post-initial FMR1 test reported	N	N	570	8 (1.4%)	98.6%	None	Not applicable	Not applicable
26	Combined workflow (≥2 methods), conventional PCR, Southern blot, other	N	PCR failure → FMRP immunodetection; Southern blot attempted but not completed.	Y	N	194	1 (0.5%)	99.5%	FMRP immunodetection	1	No additional positives
27	Conventional PCR, Southern blot, TP-PCR + capillary electrophoresis, methylation-specific PCR, combined workflow (≥2 methods)	N	Historical Southern blot diagnosis → RT-PCR reassessment ± methylation/XCI analysis.	N	Y	7	7 (100.0%)	0.0%	TP-PCR, methylation assay	7	No additional positives
28	Conventional PCR	N	PCR → Southern blot for affected or suspected PCR results.	Y	Y	114 + cascade-tested family members	9 (% not reported)	Not reported	Southern blot	Not reported	No additional positives
29	Combined workflow (≥2 methods), TP-PCR + capillary electrophoresis, Southern blot	N	FP-PCR/CE + AmplideX PCR/CE in parallel → Southern blot for positive, discordant, and selected negative cases.	Y	Y	1690	162 (9.6%)	90.4%	Southern blot	243	No additional positives
30	PCR + Southern blot	N	PCR → Southern blot when two distinct PCR products were absent.	Y	N	3488	Not reported	Not reported	Southern blot	Not reported	Not reported
31	Combined workflow (≥2 methods), PCR + Southern blot, conventional PCR	N	Blood spot PCR → peripheral blood PCR + Southern blot for positive results.	N	Y	3042	14 (0.5%)	99.5%	Southern blot, PCR	14	No additional positives
32	TP-PCR	N	dTP-PCR → FastFrax FMR1 sizing for expansion-positive samples; two mothers subsequently cascade-tested.	N	Y	54	5 (9.3%)	90.7%	PCR	3	No additional positives
33	PCR + Southern blot	N	PCR → Southern blot for all samples.	N	Y	119,232	2567 (2.2%)	97.8%	Southern blot	114,864	Not reported
34	PCR + Southern blot	N	PCR → Southern blot for failed/suspicious male results; pooled female positives retested individually.	Y	Y	575	12 (2.1%)	97.9%	Southern blot	Not reported	Not reported

**Note:** *AmplideX, commercial PCR-based assay for FMR1 CGG-repeat sizing; CAFXS, comprehensive analysis of Fragile X syndrome; CE, capillary electrophoresis; dTP-PCR, direct triplet-primed polymerase chain reaction; FM, full mutation; FMRP, fragile X messenger ribonucleoprotein; FP-PCR, fluorescent polymerase chain reaction; LRS, long-read sequencing; MS-MLPA, methylation-specific multiplex ligation-dependent probe amplification; MS-PCR, methylation-specific polymerase chain reaction; msTP-PCR, methylation-specific triplet-primed polymerase chain reaction; PCR, polymerase chain reaction; PM, premutation; SMRT, single-molecule real-time sequencing; TP-PCR, triplet-primed polymerase chain reaction; WGS, whole-genome sequencing; XCI, X-chromosome inactivation. Post-initial testing could serve one or more purposes: positive-result verification, complementary FMR1 characterization, resolution of an inconclusive result, or alternative genetic investigation after a negative result. Categories were not mutually exclusive.*

**Table 4:** Genetic counseling, management recommendations, and study limitations.

Code	Genetic Counseling	Genetic Counseling Status	Management Recommendation(s)	Limitations
1	Y	Recommendation/Benefits	Early Diagnosis/Early Intervention + Genetic Counseling	Not reported
2	Y	Recommendation/Benefits	Multidisciplinary Management	Study Design Limitations; Missing Clinical or Family Data
3	Y	Recommendation/Clinical Data Support	Newborn Screening	Small Sample Size; Limited Generalizability; Selection/Referral/Participation Bias; Study Design Limitations
4	Y	Recommendation/Benefits	Molecular Diagnosis/Risk Stratification	Technical/Assay Limitations
5	Y	Recommendation/Benefits	Early Diagnosis/Early Intervention	Not reported
6	N	Not applicable	*Not reported*	Not reported
7	Y	Clinical Implementation	Multidisciplinary Management + Genetic Counseling	Limited Generalizability
8	Y	Clinical Implementation	Multidisciplinary Management + Cascade Testing/Family Screening	Not reported
9	Y	Recommendation/Benefits	Early Diagnosis/Early Intervention + Genetic Counseling	Selection/Referral/Participation Bias; Limited Generalizability
10	Y	Recommendation/Benefits	Genetic Counseling	Missing Clinical or Family Data; Limited Generalizability
11	Y	Recommendation/Benefits	Molecular Diagnosis/Risk Stratification + Genetic Counseling	Technical/Assay Limitations
12	Y	Recommendation/Benefits	Molecular Diagnosis/Risk Stratification + Genetic Counseling	Small Sample Size; Limited Generalizability; Technical/Assay Limitations
13	Y	Recommendation/Benefits	Diagnostic Infrastructure/Standardization + Genetic Counseling	Technical/Assay Limitations; Missing Clinical or Family Data
14	Y	Recommendation/Benefits	Cascade Testing/Family Screening + Genetic Counseling	Not reported
15	Y	Recommendation/Benefits	Molecular Diagnosis/Risk Stratification	Technical/Assay Limitations
16	Y	Clinical Implementation	Early Diagnosis/Early Intervention	Technical/Assay Limitations
17	Y	Recommendation/Benefits	Molecular Diagnosis/Risk Stratification + Genetic Counseling	Small Sample Size; Selection/Referral/Participation Bias; Technical/Assay Limitations
18	Y	Recommendation/Benefits	Early Diagnosis/Early Intervention + Genetic Counseling	Missing Clinical or Family Data
19	Y	Clinical Implementation	Diagnostic Infrastructure/Standardization + Genetic Counseling	Missing Clinical or Family Data; Technical/Assay Limitations
20	N	Not applicable	*Not reported*	Selection/Referral/Participation Bias; Missing Clinical or Family Data; Technical/Assay Limitations
21	Y	Recommendation/Benefits	Early Diagnosis/Early Intervention + Genetic Counseling	Technical/Assay Limitations
22	Y	Clinical Implementation	Diagnostic Infrastructure/Standardization + Genetic Counseling	Small Sample Size; Limited Generalizability
23	Y	Clinical Implementation	Diagnostic Infrastructure/Standardization	Technical/Assay Limitations
24	Y	Recommendation/Benefits	Early Diagnosis/Early Intervention + Genetic Counseling	Not reported
25	Y	Recommendation/Benefits	Molecular Diagnosis/Risk Stratification + Multidisciplinary Management	Not reported
26	Y	Recommendation/Benefits	Newborn Screening + Cascade Testing/Family Screening + Genetic Counseling	Technical/Assay Limitations
27	Y	Clinical Implementation	Molecular Diagnosis/Risk Stratification + Genetic Counseling	Technical/Assay Limitations
28	Y	Clinical Implementation	Cascade Testing/Family Screening + Genetic Counseling	Selection/Referral/Participation Bias; Limited Generalizability
29	Y	Recommendation/Benefits	Genetic Counseling	Technical/Assay Limitations
30	Y	Recommendation/Benefits	Molecular Diagnosis/Risk Stratification + Genetic Counseling	Not reported
31	Y	Recommendation/Clinical Data Support	Early Diagnosis/Early Intervention + Cascade Testing/Family Screening + Genetic Counseling + Multidisciplinary Management	Not reported
32	Y	Recommendation/Benefits	Early Diagnosis/Early Intervention + Genetic Counseling	Limited Generalizability; Small Sample Size
33	Y	Recommendation/Clinical Data Support	Molecular Diagnosis/Risk Stratification	Not reported
34	Y	Clinical Implementation	Cascade Testing/Family Screening + Genetic Counseling	Not reported

## Data Availability

The data presented in this study are included in the article and Supplementary Materials.

## Supplementary Materials

The following supporting information can be downloaded at https://www.mdpi.com/article/10.3390/children13101338/s1. Supplementary Annex SI: Search Strategies; Supplementary Annex SII: Narrative Summary of Risk-of-Bias Assessment; Supplementary Annex SIII: Exploratory Appraisal of Evidence Strength; Supplementary Table S1: Study-level summary of risk-of-bias assessment; Supplementary Table S1a–e: Design-specific risk-of-bias assessments; Supplementary Table S2: Exploratory Evidence Strength Profile; Supplementary Table S3: Comparison with Previous FXS Reviews.
