# Supplementary material for: Advancing Standardization in Fragile X Syndrome Molecular Diagnosis: A Dual-Structured and Scoping Review
Source: Children (Basel). 2026 Oct 1;13(10):1338. doi: 10.3390/children13101338 (PMC13703091; doi:10.3390/children13101338)
Supplement: Supplementary file 1 [file children-13-01338-s001.zip › children-4552785-supplementary.pdf]

Supplementary Annex SI - Search Strategies

PubMed (MEDLINE)

("Fragile X Syndrome"[Mesh] OR "fragile X syndrome"[tiab] OR "FXS"[tiab] OR "FMR1"[tiab]) AND ("Diagnosis"[Mesh] OR "diagnostic pathways"[tiab] OR "diagnostic delay"[tiab] OR "early diagnosis"[tiab] OR screening [Mesh] OR screening[tiab] OR "case finding"[tiab] OR "referral"[tiab]) AND ("Genetic Testing"[Mesh] OR "genetic testing"[tiab] OR "molecular testing"[tiab] OR "PCR"[tiab] OR "Southern blot"[tiab] OR "methylation"[tiab] OR "CGG repeat"[tiab] OR "next-generation sequencing"[tiab] OR "NGS"[tiab]) AND ("Patient Care Management"[Mesh] OR health care delivery [Mesh] OR "management"[tiab] OR "care model"[tiab] OR "clinical pathway"[tiab] OR "follow-up"[tiab] OR "genetic counseling"[Mesh] OR "genetic counseling"[tiab] OR "access to care"[tiab] OR "health services accessibility"[Mesh] OR "barriers"[tiab]) AND ("Child"[Mesh] OR "Adolescent"[Mesh] OR "pediatric"[tiab] OR "children"[tiab] OR "adolescents"[tiab])

EMBASE (Elsevier)

('fragile x syndrome'/exp OR 'fragile x syndrome':ti,ab OR fxs:ti,ab OR fmr1:ti,ab) AND ('diagnosis'/exp OR 'early diagnosis'/exp OR 'screening'/exp OR 'diagnostic delay':ti,ab OR 'diagnostic pathway*':ti,ab OR screening:ti,ab OR referral:ti,ab) AND ('genetic testing'/exp OR 'molecular diagnosis'/exp OR 'polymerase chain reaction'/exp OR 'southern blotting'/exp OR 'dna methylation'/exp OR 'trinucleotide repeat expansion'/exp OR 'next generation sequencing'/exp OR 'genetic testing':ti,ab OR pcr:ti,ab OR 'southern blot':ti,ab OR methylation:ti,ab OR 'cgg repeat*':ti,ab OR ngs:ti,ab) AND ('health care delivery'/exp OR 'patient care'/exp OR 'disease management'/exp OR 'genetic counseling'/exp OR 'health care access'/exp OR 'health care utilization'/exp OR management:ti,ab OR 'care model*':ti,ab OR 'clinical pathway*':ti,ab OR follow-up:ti,ab OR 'genetic counseling':ti,ab OR barrier*:ti,ab) AND ('child'/exp OR 'adolescent'/exp OR pediatric:ti,ab OR child*:ti,ab OR adolescen*:ti,ab)

Scopus

TITLE-ABS-KEY ("fragile X syndrome" OR FXS OR FMR1) AND TITLE-ABS-KEY (diagnosis OR "diagnostic pathway" OR "diagnostic pathways" OR "diagnostic delay" OR "early diagnosis" OR screening OR "case finding" OR referral) AND TITLE-ABS-KEY ("genetic testing" OR "molecular testing" OR "molecular diagnosis" OR PCR OR "Southern blot" OR methylation OR "CGG repeat" OR "CGG repeats" OR "trinucleotide repeat" OR "trinucleotide repeats" OR "next generation sequencing" OR "next-generation sequencing" OR NGS) AND TITLE-ABS-KEY (management OR "care model" OR "care models" OR "clinical pathway" OR "clinical pathways" OR "follow-up" OR "follow up" OR "genetic counseling" OR "genetic counselling" OR "access to care" OR "health care access" OR "healthcare access" OR "health service" OR "health services" OR barrier OR barriers) AND TITLE-ABS-KEY (pediatric OR paediatric OR child OR children OR adolescent OR adolescents)

Supplementary Annex SII. Narrative Summary of Risk-of-Bias Assessment

Risk of bias was appraised using design-specific critical appraisal tools and summarized narratively. Because several studies presented overlapping methodological features (e.g., pilot, retrospective, service-based, laboratory comparison, or diagnostic validation components), each study was assigned a primary design according to the design most directly aligned with its principal objective and analytical structure. Cross-sectional studies were appraised using the revised JBI Critical Appraisal Tool for Analytical Cross-sectional Studies; cohort studies using the JBI Critical Appraisal Checklist for Cohort Studies; case reports using the JBI Critical Appraisal Checklist for Case Reports; and case series using the JBI Critical Appraisal Checklist for Case Series. Diagnostic accuracy and validation studies were appraised using QUADAS-2. QUADAS-2 was also applied to laboratory/method-comparison studies when the evaluated molecular assay or diagnostic approach was intended to classify *FMR1* mutation status and was assessed using established molecular methods, characterized reference materials, or other diagnostic comparators.

For the JBI tools, individual criteria were rated as Yes, No, Unclear, or Not applicable and related items were synthesized into methodologically relevant domains. QUADAS-2 signalling questions were synthesized within its four predefined domains. Domain-level risk of bias was reported as low, high, or unclear according to the signalling-question responses. No numerical score, percentage-based threshold, or overall high/moderate/low methodological-quality classification was applied. Methodological appraisal informed interpretation of the evidence rather than study eligibility, and no study was excluded on the basis of risk-of-bias findings.

Across the included studies, risk-of-bias concerns were concentrated in specific methodological domains rather than uniformly across all aspects of study conduct. The most recurrent concerns involved confounding in cross-sectional studies, patient selection and participant flow in diagnostic and laboratory studies, and completeness of follow-up or diagnostic verification. Measurement, diagnostic ascertainment, and statistical analysis were generally less problematic, although individual studies showed additional concerns in these domains. Detailed domain-level judgments are presented in Supplementary Tables S1a–e.

Cross-sectional studies.

Among the 20 studies appraised using the JBI analytical cross-sectional tool, risk of bias was generally low for selection and allocation, exposure classification, statistical analysis, and reporting. The principal methodological concern was confounding, which was judged at high risk of bias in 17 studies, while four studies also showed high risk related to outcome measurement and one to statistical conclusion validity. No domains were judged unclear. All studies were retained, as methodological appraisal was used to inform interpretation rather than eligibility.

Cohort studies

One study (code 3) was appraised using the JBI Cohort checklist. Bailey et al. showed low risk of bias for selection and exposure measurement, confounding, outcome assessment and temporality, and statistical analysis. The principal concern involved follow-up, which was judged at high risk of bias because follow-up was incomplete and strategies to address incomplete follow-up were not reported. The study was retained, as methodological appraisal was used to inform interpretation rather than study eligibility.

Case report and case series studies

One study (code 18) was appraised using the JBI Case Report checklist. Kadam et al. showed low risk of bias for patient description and history, diagnostic assessment, and reporting of takeaway lessons. Risk of bias related to intervention and follow-up was unclear, reflecting insufficient reporting of the post-intervention clinical condition. The study was retained, with appraisal findings used to inform interpretation rather than eligibility.

The two case-series studies (code 27 and 31) were appraised using the JBI Case Series checklist. Both showed low risk of bias for condition ascertainment and reporting/follow-up. Selection and inclusion were judged unclear for Roche-Martínez et al. because completeness of inclusion could not be established, and high for Sorensen et al. because consecutive and complete inclusion were not demonstrated. Statistical analysis was not applicable to either case series.

Diagnostic accuracy and laboratory/method-comparison studies

Ten studies were appraised using QUADAS-2. Risk-of-bias concerns were most evident in-patient selection and flow and timing. Patient selection was judged at high risk in four studies and unclear in four, largely reflecting non-consecutive or selected samples and incomplete reporting of recruitment. Flow and timing was at high risk in five studies, primarily because not all participants received the same reference standard or were included in the final analysis. Index-test risk was generally low, while reference-standard judgments were either low or unclear, with uncertainty mainly arising from incomplete reporting of blinding between index and reference procedures. No percentage-based overall quality ratings were calculated.

Overall risk-of-bias considerations

Across study designs, methodological concerns varied according to study design and purpose. Cross-sectional evidence was particularly affected by inadequate identification or management of confounding, whereas diagnostic accuracy and laboratory/method-comparison studies showed more frequent concerns related to patient selection and flow or reference-standard verification. Follow-up was the principal concern in the cohort study, while selection and completeness of inclusion affected the case-series evidence. These limitations should be considered alongside the frequent use of selected, referral-based, or specialist populations, which may also restrict generalizability. Overall, the appraisal supports interpretation of the evidence according to the specific domains in which bias may arise rather than through a single numerical or study-level quality score. A study-level summary is provided in Supplementary Table S1, with detailed domain-level judgments in Supplementary Tables S1a–e.

Supplementary Annex SIII – Exploratory Appraisal of Evidence Strength

The exploratory appraisal provided a contextual interpretation of the strength and limitations of the mapped evidence. The resulting evidence-strength profile (Supplementary Table S2) indicated low–moderate to moderate strength of evidence across the key outcomes. The appraisal considered risk of bias, inconsistency, indirectness, imprecision, and publication bias as an exploratory framework and was not intended to constitute a formal GRADE certainty-of-evidence assessment. Risk of bias reflected the design-specific appraisal findings; inconsistency reflected variation across populations, testing approaches, and findings; indirectness reflected the applicability of the evidence to the review questions; imprecision considered sample size, number of contributing studies, and uncertainty of estimates; and publication bias reflected the potential selective availability of findings. Overall ratings were determined by considering these domains collectively rather than through formal GRADE downgrading procedures.

The evaluated outcomes comprised *FMR1* molecular detection and diagnostic yield, use of post-initial *FMR1* testing, associations of testing method and testing setting with post-initial testing, and genetic counseling and management recommendations. Evidence for molecular detection and post-initial testing was rated moderate, supported by findings across multiple study designs and populations, although heterogeneity in recruitment, clinical indication, testing methods, and post-initial testing workflows limited comparability. Evidence concerning genetic counseling and management recommendations was also rated moderate, with counseling addressed in 32 of 34 studies and explicit clinical implementation documented in 9 of 34; however, most studies described recommendations or anticipated benefits rather than comparative management outcomes.

Evidence for the associations between post-initial *FMR1* testing and genetic testing method and testing setting was rated low–moderate. Genetic testing method was associated with post-initial testing ($p = 0.005$), whereas testing setting showed a borderline nominal association ($p = 0.046$). These analyses were exploratory, based on study-level categorical data, and unadjusted for multiple comparisons; the testing-setting analysis was additionally limited by small category counts. The evidence base included several large clinical, laboratory, and screening cohorts, while heterogeneity in study populations and diagnostic workflows and domain-specific risk-of-bias concerns remained principal limitations to confidence and comparability across studies.

Supplementary Table S1. Study-level summary of risk-of-bias assessment

Code	Study ID	Primary Study Design	Appraisal Tool	Key Concerns
1	Alliende et al., 2008 [48]	Cross-sectional	JBI Cross-sectional	School-based sample; cross-sectional design; findings may not generalize beyond special-education populations.
2	Au et al., 2025 [8]	Cross-sectional	JBI Cross-sectional	Retrospective design; phenotype analysis limited to confirmed cases; no structured long-term follow-up.
3	Bailey et al., 2017 [49]	Cohort	JBI Cohort	Voluntary screening; incomplete follow-up; possible participation bias.
4	Bhowmik et al., 2009 [50]	Cross-sectional	JBI Cross-sectional	Single-center cross-sectional study; small number of confirmed FXS cases; limited longitudinal outcome data.
5	Chow et al., 2003 [51]	Cross-sectional	JBI Cross-sectional	Screening study without confirmatory follow-up of identified candidates; newborn males only.
6	Christofolini et al., 2007 [52]	Diagnostic Accuracy / Validation	QUADAS-2	Male-only tertiary outpatient sample; checklist validation restricted to individuals with normal karyotypes.
7	Cotter et al., 2016 [53]	Cross-sectional	JBI Cross-sectional	Administrative laboratory database; irregular follow-up; limited clinical detail beyond testing records.
8	Dave & Shetty, 2010 [54]	Laboratory / Method comparison	QUADAS-2	Substantial attrition between screening and molecular analysis cohorts; referral-based sample; limited generalizability.
9	Fatima et al., 2014 [55]	Cross-sectional	JBI Cross-sectional	School-based sample with incomplete school participation and potential selection bias; limited generalizability.
10	Garrido-Torres et al., 2024 [56]	Cross-sectional	JBI Cross-sectional	Retrospective ASD cohort; referral-based sample; limited generalizability.
11	Gigonzac et al., 2016 [57]	Laboratory / Method comparison	QUADAS-2	Very small sample (N=26); laboratory implementation study; no follow-up or clinical outcome assessment.
12	González-del Angel et al., 2000 [58]	Cross-sectional	JBI Cross-sectional	Small tertiary-referral sample; limited generalizability; confounding not addressed.
13	Hnoonual et al., 2025 [59]	Cross-sectional	JBI Cross-sectional	Single-country referral cohort; follow-up inconsistently reported; laboratory-based rather than population-based sample.
14	Hosny Omar et al., 2016 [60]	Cross-sectional	JBI Cross-sectional	Small clinically selected male-only cohort; cross-sectional design; potential overestimation of diagnostic yield.
15	Hou et al., 2023 [61]	Laboratory / Method comparison	QUADAS-2	Moderate sample size; laboratory validation study; limited clinical outcome and follow-up data.
16	Ibañez et al., 2022 [62]	Diagnostic Accuracy / Validation	QUADAS-2	Highly selected diagnostic cohort; focused on repeat-expansion disorders; limited direct Fragile X-specific applicability.
17	Jain et al., 2013 [63]	Cross-sectional	JBI Cross-sectional	Single-center tertiary-care cohort; modest sample size; referral-based population may not represent community cases.
18	Kadam et al., 2018 [64]	Case report	JBI Case Report	Very small familial case report (N=2 siblings); highly selected family presentation; findings not generalizable.
19	Kamga et al., 2020 [65]	Cross-sectional	JBI Cross-sectional	Very small family-based sample (N=46); highly selected pedigree; limited generalizability despite detailed cascade testing.
20	Kraan et al., 2018 [66]	Cross-sectional	JBI Cross-sectional	Cross-sectional prevalence study; referral-based cohorts; limited clinical phenotyping and longitudinal follow-up.
21	Meguid et al., 2014 [67]	Laboratory / Method comparison	QUADAS-2	Clinically selected sample from affected families; screening assay unable to reliably detect full mutations or mosaicism.
22	Penteado et al., 2017 [68]	Cross-sectional	JBI Cross-sectional	Small retrospective referral sample; limited generalizability; confounding not addressed.
23	Pozo-Palacios et al., 2021 [69]	Cross-sectional	JBI Cross-sectional	Clinically selected male-only sample; cross-sectional design; endpoint PCR limits diagnostic classification.
24	Rajan-Babu et al., 2022 [70]	Diagnostic Accuracy / Validation	QUADAS-2	Laboratory validation study with modest sample size; limited clinical validation beyond reference cohorts.
25	Rauch et al., 2006 [71]	Laboratory / Method comparison	QUADAS-2	Referral-based genetics cohorts; heterogeneous diagnostic pathways; limited follow-up information.
26	Rifé et al., 2002 [72]	Cross-sectional	JBI Cross-sectional	Small pilot screening study; confirmatory testing incomplete for the probable affected case; limited sample size.
27	Roche-Martínez et al., 2025 [73]	Case series	JBI Case Series	Case-series design; highly selected atypical cases; limited generalizability despite detailed longitudinal assessment.
28	Ruangdaraganon et al., 2000 [74]	Cross-sectional	JBI Cross-sectional	Single-clinic selected developmental-delay sample; 15/129 eligible children declined testing; confounding not addressed.
29	Shou et al., 2025 [75]	Diagnostic Accuracy / Validation	QUADAS-2	Few excluded samples; referral-based testing population; diagnostic accuracy study without long-term clinical follow-up
30	Sofocleous et al., 2008 [76]	Cross-sectional	JBI Cross-sectional	Retrospective referral-center cohort; family studies may overrepresent inherited cases; limited follow-up standardization.
31	Sorensen et al., 2013 [77]	Case series	JBI Case Series	Voluntary screening program; small number of cascade-tested relatives; limited long-term outcome assessment.
32	Stoyanova et al., 2022 [78]	Cross-sectional	JBI Cross-sectional	Small, clinically selected high-risk sample; predominantly male and hospital-based; confounding not addressed.
33	Strom et al., 2007 [79]	Cross-sectional	JBI Cross-sectional	Laboratory database study; no clinical follow-up; limited phenotypic and outcome information.
34	Tzeng et al., 2001 [80]	Diagnostic Accuracy / Validation	QUADAS-2	Screening-focused validation study; no follow-up; potential spectrum bias from clinically selected participants.

Supplementary Table S1a. JBI Risk-of-Bias Assessment for Analytical Cross-sectional Studies

Code	Selection & allocation (Q1–Q2)	Exposure classification (Q3)	Outcome measurement (Q4)	Confounding (Q5–Q6)	Statistical conclusion validity (Q7)	Reporting (Q8)
1	Low	Low	Low	High	Low	Low
2	Low	Low	Low	High	Low	Low
4	Low	Low	High	High	Low	Low
5	Low	Low	High	High	Low	Low
7	Low	Low	Low	Low	Low	Low
9	Low	Low	Low	High	Low	Low
10	Low	Low	Low	Low	Low	Low
12	Low	Low	Low	High	Low	Low
13	Low	Low	Low	High	Low	Low
14	Low	Low	Low	High	Low	Low
17	Low	Low	Low	High	Low	Low
19	Low	Low	Low	High	Low	Low
20	Low	Low	Low	Low	Low	Low
22	Low	Low	Low	High	Low	Low
23	Low	Low	High	High	High	Low
26	Low	Low	High	High	Low	Low
28	Low	Low	Low	High	Low	Low
30	Low	Low	Low	High	Low	Low
32	Low	Low	Low	High	Low	Low
33	Low	Low	Low	High	Low	Low

Note: The revised JBI Critical Appraisal Tool for Analytical Cross-sectional Studies organizes signaling questions into domains relating to selection and allocation (Q1–Q2), classification of exposure (Q3), assessment/detection/measurement of outcomes (Q4), confounding (Q5–Q6), and statistical conclusion validity (Q7); Q8 assesses comprehensiveness of reporting. Domain-level risk of bias was classified as low when all relevant signaling questions were rated "Yes," high when one or more were rated "No," and unclear when one or more were rated "Unclear" in the absence of a "No" judgment. No numerical score or percentage-based overall quality rating was calculated.

Supplementary Table S1b. JBI Risk-of-Bias Assessment for Cohort Studies

Code	Selection & exposure measurement (Q1–Q3)	Confounding (Q4–Q5)	Outcome assessment & temporality (Q6–Q8)	Follow-up (Q9–Q10)	Statistical analysis (Q11)
3	Low	Low	Low	High	Low

Note: The JBI Cohort Checklist assessed 11 criteria rated as Yes, No, Unclear, or Not applicable. For synthesis, items were grouped into selection/exposure measurement (Q1–Q3), confounding (Q4–Q5), outcome assessment/temporality (Q6–Q8), follow-up (Q9–Q10), and statistical analysis (Q11). Risk of bias was rated low when all relevant items were "Yes," high when any were "No," and unclear when one or more were "Unclear" with no "No" ratings. No numerical or percentage-based overall score was used.

Supplementary Table S1c. JBI Risk-of-Bias Assessment for Case Reports

Code	Patient description & history (Q1–Q3)	Diagnostic assessment (Q4)	Intervention & follow-up (Q5–Q7)	Takeaway lessons (Q8)
18	Low	Low	Unclear	Low

Note: The JBI Critical Appraisal Checklist for Case Reports assessed eight criteria rated as Yes, No, Unclear, or Not applicable. For synthesis, related items were grouped into patient description and history (Q1–Q3), diagnostic assessment (Q4), intervention and follow-up (Q5–Q7), and takeaway lessons (Q8). Domain-level risk of bias was classified as low when all relevant items were rated "Yes," high when one or more were rated "No," and unclear when one or more were rated "Unclear" in the absence of a "No" judgment. No numerical score or percentage-based overall quality rating was calculated.

Supplementary Table S1d. JBI Risk-of-Bias Assessment for Case Series

Code	Selection & inclusion (Q1, Q4–Q5)	Condition ascertainment (Q2–Q3)	Reporting & follow-up (Q6–Q9)	Statistical analysis (Q10)
27	Unclear	Low	Low	N/A
31	High	Low	Low	N/A

Note: The JBI Case Series Checklist assessed 10 criteria rated as Yes, No, Unclear, or Not applicable. For synthesis, items were grouped into selection/inclusion (Q1, Q4–Q5), condition ascertainment (Q2–Q3), reporting/follow-up (Q6–Q9), and statistical analysis (Q10). Risk of bias was rated low when all relevant items were "Yes," high when any were "No," and unclear when one or more were "Unclear" with no "No" ratings. No numerical or percentage-based overall score was used.

Supplementary Table S1e. QUADAS-2 Risk-of-Bias Assessment

Code	Patient Selection (Q1–Q3)	Index Test (Q4–Q5)	Reference Standard (Q6–Q7)	Flow & Timing (Q8–Q11)
6	Unclear	High	Unclear	Low
8	High	Unclear	Unclear	High
11	Unclear	Low	Low	High
15	High	Low	Unclear	Low
16	High	Low	Unclear	Low
21	Low	N/A	Low	High
24	High	Low	Low	High
25	Low	N/A	Low	Low
29	Unclear	Low	Unclear	Low
34	Unclear	Unclear	Unclear	High

Note: QUADAS-2 assesses risk of bias across four domains: patient selection (Q1–Q3), index test (Q4–Q5), reference standard (Q6–Q7), and flow and timing (Q8–Q11). Domain judgments were rated as low, high, or unclear risk of bias according to the signalling-question responses; N/A indicates that the relevant questions were not applicable. No numerical or percentage-based overall quality score was used.

Supplementary Table S2. Exploratory Evidence Strength Profile

Outcome	Study Designs Contributing	Risk of Bias	Inconsistency	Indirectness	Imprecision	Publication Bias	Overall strength of evidence	Comments
FMR1 molecular detection and diagnostic yield	Cross-sectional + diagnostic/validation + laboratory comparison + cohort	Low–Moderate	Moderate	Low–Moderate	Low–Moderate	Unclear	Moderate	Molecular abnormalities were consistently detected across diverse populations and methods; variation in yield largely reflected differences in recruitment, clinical indication, and testing workflow.
Use of post-initial FMR1 testing	Cross-sectional + diagnostic/validation + laboratory comparison + cohort	Low–Moderate	Moderate	Low	Low	Unclear	Moderate	Post-initial FMR1 testing was reported in 22/34 studies (64.7%); approaches and purposes varied across studies.
Testing method associated with post-initial FMR1 testing	Cross-study exploratory analysis	Moderate	Low–Moderate	Low	Moderate	Unclear	Low–Moderate	Post-initial testing differed by genetic testing method ($p=0.005$); analysis was exploratory and based on 34 studies.
Testing setting associated with post-initial FMR1 testing	Cross-study exploratory analysis	Moderate	Low–Moderate	Low	Moderate	Unclear	Low–Moderate	Testing setting showed a borderline nominal association with post-initial testing ($p=0.046$); the finding should be interpreted cautiously given small category counts and the absence of adjustment for multiple comparisons.
Genetic counseling and management recommendations	Cross-sectional + cohort + diagnostic/validation + laboratory comparison + case-based evidence	Low–Moderate	Low–Moderate	Moderate	Low–Moderate	Unclear	Moderate	Genetic counseling was addressed in 32/34 studies, with explicit clinical implementation documented in 9/34 (26.5%); most evidence concerned recommendations or anticipated benefits rather than comparative management outcomes.

Note: This profile represents an exploratory, descriptive appraisal rather than a formal GRADE certainty-of-evidence assessment. Domain and overall ratings were assigned using the decision framework described in Supplementary Annex III.

Supplementary Table S3. Comparison with Previous FXS Reviews

Previous Review	Scope / Population	Geographic Scope	Molecular testing / characterization	Complementary testing when indicated	Genetic counseling / family testing	Multidisciplinary management	Overall alignment	Main finding relevant to this review
Abrams et al., 2012 [82]	FMR1 newborn, carrier, and early-childhood screening in children and families.	Multinational, predominantly North America	Aligned — direct FMR1 testing	Partial — no clear confirmatory algorithm	Aligned — counseling + cascade testing	Aligned — early intervention + coordinated care	High	Earlier identification supports counseling, family testing, and intervention.
Mendez & Mendez, 2024 [86]	Pediatric FXS recognition, diagnosis, counseling, and management.	North America (USA)	Aligned — direct FMR1 testing	Partial — no clear confirmatory algorithm	Aligned — counseling + family testing	Aligned — multidisciplinary care	High	Molecular diagnosis should lead to counseling, family testing, and coordinated care.
Okoniewski et al., 2019 [84]	Early identification and newborn screening, including the Early Check program.	North America (USA)	Aligned — FMR1 expansion testing	Aligned — confirmatory characterization	Aligned — counseling + family testing	Aligned — developmental follow-up + intervention	High	Screening can connect molecular confirmation with counseling and developmental follow-up.
Pandey et al., 2004 [81]	Molecular diagnosis and genetic counseling in affected individuals and families.	Asia (India)	Aligned — PCR/Southern blot characterization	Aligned — complementary testing when needed	Aligned — counseling + carrier testing	Partial — management discussed but not structured	High	Molecular diagnosis enables complementary testing, family evaluation, and counseling.

Previous Review	Scope / Population	Geographic Scope	Molecular testing / characterization	Complementary testing when indicated	Genetic counseling / family testing	Multidisciplinary management	Overall alignment	Main finding relevant to this review
Protic et al., 2022 [87]	FXS diagnosis and treatment across the lifespan.	Multinational / Global	Aligned — molecular FMR1 characterization	Aligned — complementary approaches	Partial — family implications addressed	Aligned — multidisciplinary intervention	High	Molecular diagnosis should be followed by individualized multidisciplinary treatment.
Savatt & Myers, 2021 [83]	Genetic testing for pediatric GDD, ID, and ASD, including FMR1 testing.	Predominantly North America	Aligned — FMR1 CGG-repeat analysis	Partial — no universal confirmatory workflow	Aligned — counseling + family implications	Aligned — diagnosis informs surveillance and care	High	FMR1 testing forms part of broader etiologic evaluation of neurodevelopmental disorders.
Tassone, 2014 [85]	Newborn screening for FXS/FMR1 variants in infants and families.	Multinational / Global	Aligned — PCR-based FMR1 screening	Partial — no clear confirmatory algorithm	Aligned — counseling + cascade testing	Aligned — intervention + long-term follow-up	High	Screening may enable earlier intervention, counseling, cascade testing, and follow-up.

Note: Alignment was assessed descriptively against the current diagnostic and management recommendations identified in the structured review. “Aligned” indicates that the review was consistent with the relevant recommendation, whereas “Partial” indicates that the domain was addressed but incompletely or without a clearly specified pathway. Overall alignment reflects the recommendations addressed by each review and does not represent a formal quality assessment.
